# Estimating Ocean Heat Uptake Using Boundary Green's Functions: A Perfect‐Model Test of the Method

**DOI:** 10.1029/2022MS002999

**Published:** 2022-12-21

**Authors:** Quran Wu, Jonathan M. Gregory

**Affiliations:** ^1^ National Centre for Atmospheric Science University of Reading Reading UK; ^2^ Met Office Hadley Centre Exeter UK

**Keywords:** ocean heat uptake, passive tracer, Green's function, maximum entropy

## Abstract

Ocean heat uptake is caused by “excess heat” being added to the ocean surface by air‐sea fluxes and then carried to depths by ocean transports. One way to estimate excess heat in the ocean is to propagate observed sea surface temperature (SST) anomalies downward using a Green's function (GF) representation of ocean transports. Taking a “perfect‐model” approach, we test this GF method using a historical simulation, in which the true excess heat is diagnosed. We derive GFs from two approaches: (a) simulating GFs using idealized tracers, and (b) inferring GFs from simulated CFCs and climatological tracers. In the model world, we find that combining simulated GFs with SST anomalies reconstructs the Indo‐Pacific excess heat with a root‐mean‐square error of 26% for depth‐integrated changes; the corresponding number is 34% for inferred GFs. Simulated GFs are inaccurate because they are coarse grained in space and time to reduce computational cost. Inferred GFs are inaccurate because observations are insufficient constraints. Both kinds of GFs neglect the slowdown of the North Atlantic heat uptake as the ocean warms up. SST boundary conditions contain redistributive cooling in the Southern Ocean, which causes an underestimate of heat uptake there. All these errors are of comparable magnitude, and tend to compensate each other partially. Inferred excess heat is not sensitive to: (a) small changes in the shape of prior GFs, or (b) additional constraints from SF_6_ and bomb ^14^C.

## Key Points

- Green's functions (GFs) broadly reconstruct simulated ocean heat uptake when constrained by simulated observations in a historical simulation
- Errors in the GF method arise from insufficient observational constraints and forced changes in ocean transports
- GFs underestimate the Southern Ocean heat uptake when using sea surface temperature anomalies as boundary conditions

## 1 Introduction

Imbalance in Earth's top‐of‐atmosphere radiative forcing leads to accumulation of “excess heat” in the climate system. Over 93% of the excess heat is stored in the ocean, causing ocean warming and sea‐level rise (Meyssignac et al., 2019). Excess heat invades the ocean from the surface, like a drop of dye spreads in a water tank. This process can be conveniently described using a mathematical tool called Green's functions (GFs). Here, we examine how accurately excess heat in the ocean can be estimated using GFs.

A change in ocean heat content can be understood in terms of excess and redistributed heat content. Excess heat is defined as the change (warming or cooling) that is added to the ocean by air‐sea fluxes, and then carried to depths by ocean transports. Redistributed heat, on the other hand, is defined as the change of the pre‐existing heat in the ocean (i.e., spatial redistribution). Isolating excess heat is useful because: (a) excess heat change can be constrained by observations of transient tracers in the ocean (see Section 6), and (b) excess heat change dominates global/basin integrated ocean heat content change. Under CO_2_ forcing, climate models show that excess heat largely accumulates in the North Atlantic and the Southern Ocean, while redistributed heat tends to accumulate at low latitudes (Gregory et al., 2016; Newsom et al., 2022). Excess and redistributed heat are both theoretical constructs; neither of them is directly observable in the ocean.

Excess heat at depths can be estimated by propagating its surface “source” downward using boundary GFs of the tracer equation (Holzer & Hall, 2000). We refer to this method as the GF method. The source or boundary condition (BC) of excess heat is often computed from observed sea surface temperature (SST) in the literature (e.g., Messias & Mercier, 2022; Zanna et al., 2019). Boundary GFs represent the ocean's surface‐to‐interior transport. They can be derived from: (a) simulating idealized tracers in a model (e.g., Khatiwala et al., 2005; Zanna et al., 2019) or (b) solving an inverse problem using tracer observations (e.g., Holzer et al., 2010; Khatiwala et al., 2009). The GF method adds useful information to the in‐situ estimate of ocean heat content change. The two estimates are directly comparable for basin integrals; regionally, their differences indicate redistributed heat in the ocean.

We refer to GFs derived from model simulations as “simulated GFs,” and GFs inferred from tracer observations as “inferred GFs.” In practice, both types of GFs are at best approximations of the real‐world ocean transport, due to various assumptions, simplifications and trade‐offs. Simulated GFs are often coarse grained in space and time, hence they do not fully capture the covariance between the true GFs and surface BCs. In addition, simulated GFs rely on a model's ocean transports, but no model is perfect. Inferred GFs, on the other hand, do not rely on a model; but they too are inaccurate, because observations are insufficient constraints.

As well as the GFs, surface BCs are not perfectly known for estimating excess heat. SST anomalies, as used by Zanna et al. (2019), are contaminated by redistributed temperatures which are not BCs of excess heat. This error affects both types of GFs.

The accuracy of the GF method for estimating excess heat in the ocean has not been examined in the literature. In this study, we address this problem using a HadCM3 historical simulation (1860–2008). We treat this simulation as the real world, and compare excess heat diagnosed in it (as the “truth”) with that estimated using simulated/inferred GFs. This approach is useful because it allows a separation of excess and redistributed heat and a quantification of different errors, both of which are not accessible in observations.

Because our historical simulation agrees well with observations for large‐scale ocean heat uptake, our error estimates are relevant to applying the GF method to the real world. Importantly, our result pinpoints the main error sources in the GF method, and provides a quantitative benchmark for each of them. Nonetheless, we expect that at least some of our error estimates are HadCM3 specific, especially since HadCM3 is a coarse resolution model and not constrained by observations. Future studies with high‐resolution models or ocean state estimates would be useful to provide a more robust error estimate.

Setup of the HadCM3 historical simulation and definitions of excess and redistributed heat are explained in Section 2. In Section 3, we explain how to solve the passive tracer equation using GFs. Section 4 explains the method of simulating GFs. Section 5 evaluates excess heat estimates based on simulated GFs. The same pattern is repeated in Sections 6 and 7, but for inferred GFs. Finally, a summary is given in Section 8 and discussions in Section 9.

## 2 Historical Simulation and Temperature Tracers

### 2.1 Setup of Historical Simulation

HadCM3 is an Atmosphere‐Ocean General Circulation Model (AOGCM) that has been used extensively for climate studies (Gordon et al., 2000). The HadCM3 atmosphere model is based on the UK Met Office Unified Model, with a horizontal resolution of 2.5° × 3.75° and 19 vertical layers. The HadCM3 ocean model is based on the Cox (1984) model with a horizontal resolution of 1.25° × 1.25° and 20 vertical levels (vertical resolution is enhanced near the surface). Horizontal eddy mixing of tracers in the HadCM3 ocean is parameterized using the Gent and Mcwilliams (1990) and Redi (1982) schemes.

We run a pre‐industrial control experiment and a historical experiment in parallel with HadCM3. Both experiments start from a pre‐industrial state at 1860 and run to 2008. (This choice omits the ocean's slow response to the global cooling before 1860 cf., Gebbie and Huybers (2019).) The historical experiment is conducted by adding historical effective radiative forcing *Q*_ERF_ (space and time dependent) to the sea‐water surface. To compute *Q*_ERF_ the atmosphere model ECHAM6.3 (Giorgetta et al., 2013) is forced with time‐dependent historical changes in all forcing agents and fixed pre‐industrial SSTs and sea ice concentrations, following the design of the piClim‐histall experiment (Pincus et al., 2016). This ECHAM6.3 simulation is used in Gregory et al. (2020) to compute the global‐mean *Q*_ERF_. Note that we choose to force HadCM3 with *Q*_ERF_ instead of adding forcing agents to its atmosphere; Appendix A explains the motivation for this choice.

### 2.2 Evolution Equations of Temperature Tracers

#### 2.2.1 Historical and Control Temperatures

Evolution of ocean potential temperature Θ in the control and historical experiments can, in general, be written as

(1) $$\begin{array}{ll} & \frac{\partial \Theta }{\partial t}+\Phi \left(\Theta \right)=\Psi ,\\ & \text{initial condition:}\Theta \left(0\right)=\Theta_{0}. \end{array}$$

Θ_0_ is the pre‐industrial state at 1860. Ψ is the source/sink of Θ; it is zero everywhere except at the surface (ignoring geothermal heat flux). Φ is the ocean transport operator that evolves an ocean tracer field forward in time. A simple form of Φ can be given as

(2) $$\Phi \left(\chi \right)=v\cdot \nabla \chi -\nabla \cdot \left(\kappa_{\delta }\nabla_{\delta }\chi \right)-\frac{\partial }{\partial z}\left(\kappa_{z}\frac{\partial \chi }{\partial z}\right).$$

*χ* is the concentration of a tracer. **v** is a 3D velocity vector. *κ*_*δ*_ and *κ*_*z*_ are isopycnal and vertical eddy diffusivities, respectively. ∇_*δ*_ computes the lateral gradient of a scalar on isopycnal surfaces. Note that the Φ operator in modern ocean models is more complex than Equation 2.

The control and historical Θ fields are different because the two experiments have different Φ and Ψ. The ocean transport operator Φ is different because global warming affects ocean transports in many ways; for example, a reduction in high‐latitude convection. The surface source Ψ = *Q*_ctrl_/(*ρ*_0_ *c*_*p*_d*z*_1_) in the control experiment, where *Q*_ctrl_ is the net surface heat flux (W m^−2^) under the pre‐industrial condition. In the historical experiment Ψ = (*Q*_ctrl_ + *Q*_ERF_ + *Q*′)/(*ρ*_0_ *c*_*p*_d*z*_1_); the two additional terms come from: (a) the historical forcing (*Q*_ERF_) and (b) climate feedbacks (*Q*′) in response to the forcing. *ρ*_0_ *c*_*p*_d*z*_1_ is the top layer thermal inertia (J K^−1^ m^−2^), wherein *ρ*_0_ is reference density, *c*_*p*_ specific heat capacity, and d*z*_1_ top layer thickness.

#### 2.2.2 Linear Equations of Temperature Evolution

Φ is a non‐linear operator when applied to Θ, because Φ itself depends on Θ; for instance, **v** in Equation 2 is a function of Θ. To facilitate a linear decomposition of temperature change, we define two linear versions of Φ, denoted as *L*_ctrl_ and *L*_hist_, using velocities and diffusivities from the control and historical experiments, respectively. Unlike the Φ operator, which is a function of ocean states, the *L* operator is a pre‐computed quantity, for example, an array of coefficients. The evolution equation of Θ in the control experiment can be rewritten as

(3) $$\begin{array}{ll} & \frac{\partial \Theta_{\text{ctrl}}}{\partial t}+L_{\text{ctrl}}\left(\Theta_{\text{ctrl}}\right)=\frac{1}{\rho_{0}c_{p}dz_{1}}Q_{\text{ctrl}},\\ & \text{initial condition:}\Theta_{\text{ctrl}}\left(0\right)=\Theta_{0}. \end{array}$$

Similarly, the evolution equation of Θ in the historical experiment can be rewritten as

(4) $$\begin{array}{ll} & \frac{\partial \Theta_{\text{hist}}}{\partial t}+L_{\text{hist}}\left(\Theta_{\text{hist}}\right)=\frac{1}{\rho_{0}c_{p}dz_{1}}\left(Q_{\text{ctrl}}+Q_{\text{ERF}}+Q^{\prime }\right),\\ & \text{initial condition:}\Theta_{\text{hist}}\left(0\right)=\Theta_{0}. \end{array}$$

It is important to note that *L*_ctrl_ and *L*_hist_ are linear operators when applied to any tracer fields. For instance, we have *L*_hist_(Θ_hist_) − *L*_hist_(Θ_ctrl_) = *L*_hist_(Θ_hist_ − Θ_ctrl_). The same does not hold for the Φ operator because it is nonlinear in Θ. In Section 2.2.3, we will use the linearity of *L*_hist_ to derive the governing equation of redistributed temperature. *L*_ctrl_ and *L*_hist_ both have time‐varying coefficients due to variability and change in ocean transports.

#### 2.2.3 Excess and Redistributed Temperatures

Temperature anomaly in the historical simulation relative to the control can be written as the sum of two passive tracers, Θ_e_ and Θ_r_

(5) $$\Theta_{a}=\Theta_{\text{hist}}-\Theta_{\text{ctrl}}=\Theta_{e}+\Theta_{r}.$$

The excess temperature Θ_e_ is the part of Θ_a_ driven by changes in surface heat fluxes (Equations 3 and 4 have different right‐hand‐side forcing terms). Its evolution equation is defined as

(6) $$\begin{array}{ll} & \frac{\partial \Theta_{e}}{\partial t}+L_{\text{hist}}\left(\Theta_{e}\right)=\frac{1}{\rho_{0}c_{p}dz_{1}}\left(Q_{\text{ERF}}+Q^{\prime }\right),\\ & \text{initial condition:}\Theta_{e}\left(0\right)=0. \end{array}$$

We implement Equation 6 by simulating Θ_e_ as a passive tracer in the historical simulation from 1860 to 2008. The redistributed temperature Θ_r_ is the part of Θ_a_ driven by changes in ocean transports (Equations 3 and 4 have different transport operators). Its evolution equation can be derived by combining Equations (3), (4), (5), (6) and making use of the linearity of *L*_hist_.

(7) $$\begin{array}{ll} & \frac{\partial \Theta_{r}}{\partial t}+L_{\text{hist}}\left(\Theta_{r}\right)=L_{\text{ctrl}}\left(\Theta_{\text{ctrl}}\right)-L_{\text{hist}}\left(\Theta_{\text{ctrl}}\right),\\ & \text{initial condition:}\Theta_{r}\left(0\right)=0. \end{array}$$

Note that changes in ocean transports acting on Θ_ctrl_ are the source term of Θ_r_. For convenience, we compute Θ_r_ as Θ_a_ − Θ_e_ (Equation 5) instead of using Equation 7. In the historical simulation, Θ_e_ and Θ_r_ are both affected by unforced variability and forced climate change. We focus on multi‐decadal changes in Θ_e_ and Θ_r_ to highlight the role of forced response.

The key difference between Θ_e_ and Θ_r_ is that Θ_e_ only comes from the surface, while Θ_r_ has sources throughout the volume of the ocean. The global volume integral of Θ_r_ is zero, because the effect of *L* integrates to zero over the global ocean. (Θ_r_ defined in Gregory et al. (2016) does not have exactly zero volume integral.)

#### 2.2.4 Converting Temperature to Heat Content

We compute ocean heat content anomaly $H_{a}$ as $\int_{V}\rho_{0}c_{p}\left(\Theta_{\text{hist}}-\Theta_{\text{ctrl}}\right)d^{3}r$. **r** is a 3D position vector of the ocean, V an arbitrary control volume, and *ρ*_0_ *c*_*p*_ ≡ 4 × 10^6^ J K^−1^ m^−3^. Applying the same procedure to Θ_e_ and Θ_r_ results in excess heat content $\left(H_{e}\right)$ and redistributed heat content $\left(H_{r}\right)$, respectively $\left(H_{a}=H_{e}+H_{r}\right)$.

### 2.3 Evaluation of Historical Simulation

**Figure 1:**
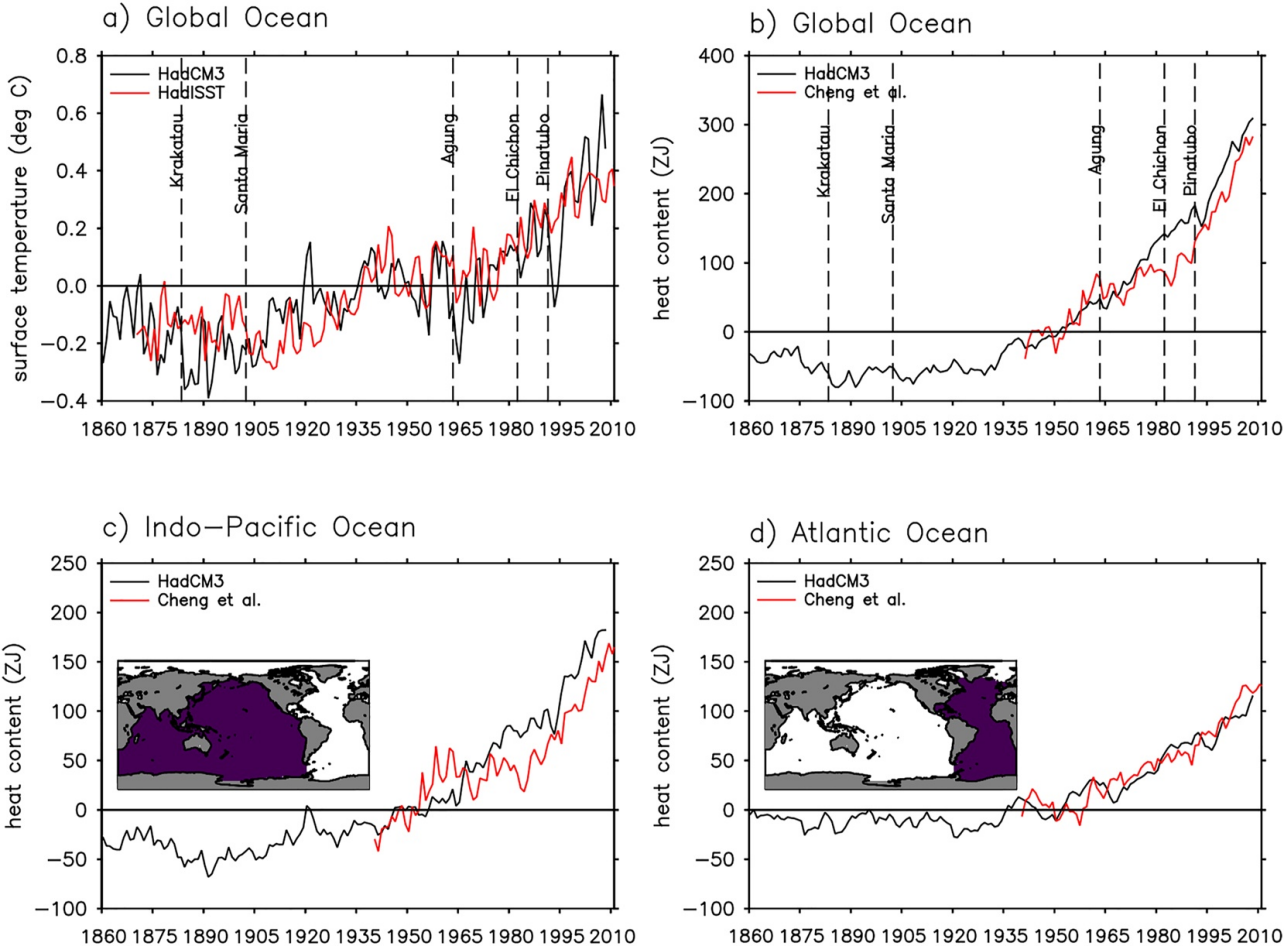
Surface and depth integrated ocean warming in the HadCM3 historical simulation. (a) Global averaged sea surface temperature. (b) Global integrated ocean heat content. Panels (c and d) are the same as panel (b), but for basin integrals. Global and basin integrals are calculated for the 0–2,000 m layers. All quantities are shown as anomalies relative to the 1946–1955 average. For comparison, observational estimates from Rayner et al. (2003) (HadISST) and Cheng et al. (2017) (heat content) are also plotted. 1 ZJ = 1 × 10^21^ J.

The HadCM3 historical simulation captures the surface and depth integrated ocean warming in observations reasonably well (Figure 1). The global mean SST in HadCM3 generally follows that in HadISST (Rayner et al., 2003), but it does not capture the early 21st century warming hiatus in HadISST (Figure 1a). HadCM3 also tends to overestimate the surface cooling after volcanic eruptions compared to observations (Figure 1a); this is a common feature among CMIP5 models (D. M. Smith et al., 2016; Marotzke & Forster, 2015). In both HadCM3 and observations of Cheng et al. (2017), the global integrated $H_{a}$ (0–2,000 m) increases by about 300 ZJ in 2008 relative to 1946–1955 (1 ZJ = 1 × 10^21^ J); more than half of that is stored in the Indo‐Pacific (Figures 1b–1d). HadCM3 does not capture the observed plateauing of $H_{a}$ increase after the 1963 Mount Agung eruption (Figures 1b and 1c).

We compare $H_{e}$ and $H_{r}$ simulated in HadCM3 with those in Bronselaer and Zanna (2020) (BZ2020). BZ2020 infers $H_{e}$ by scaling the pattern of anthropogenic carbon in the ocean; $H_{r}$ is then derived by subtracting inferred $H_{e}$ from observed $H_{a}$ change.

**Figure 2:**
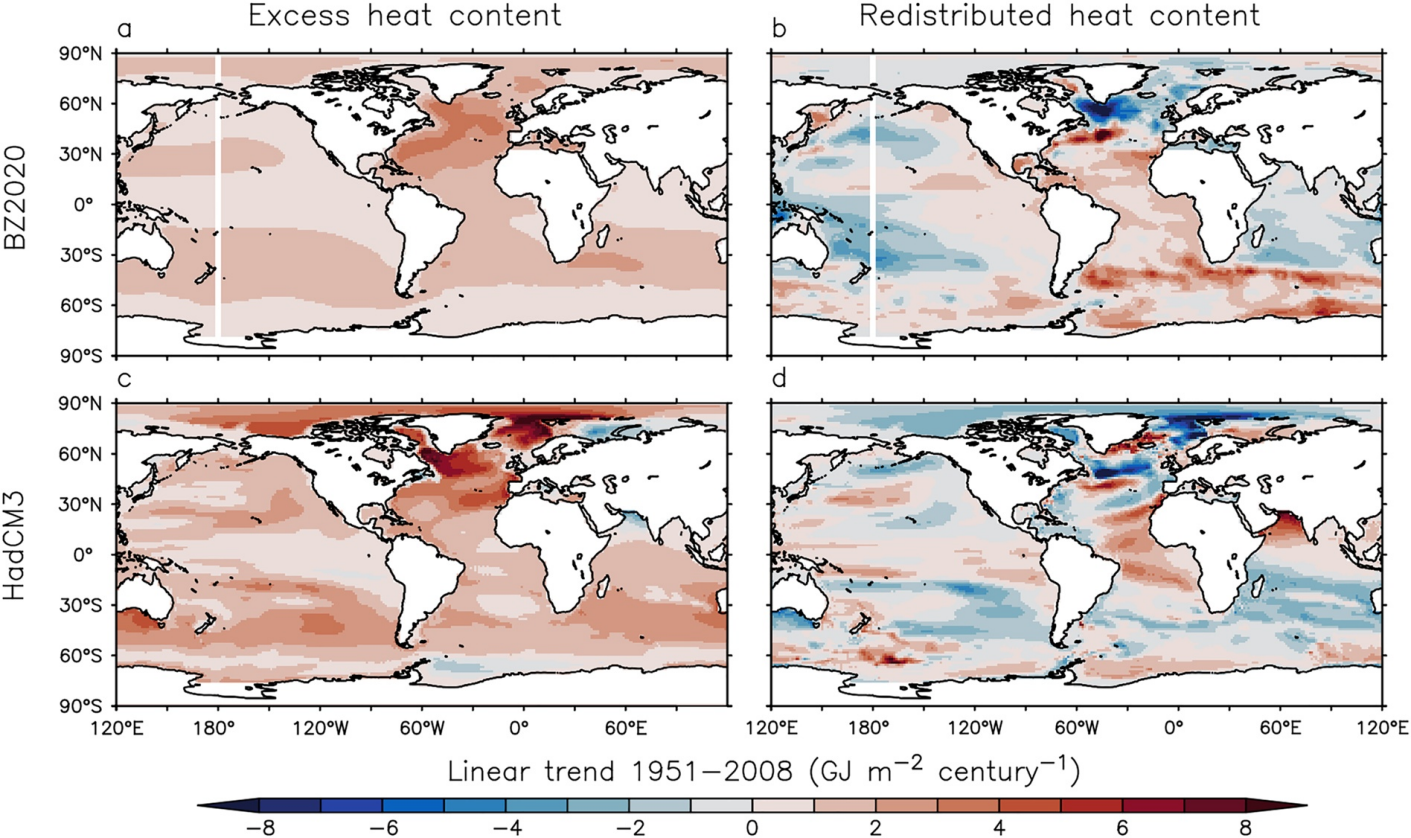
Linear trends of excess and redistributed heat content (0–2,000 m integrated) during the 1951–2008 period. (a and b) Bronselaer and Zanna (2020) (based on anthropogenic carbon). (c and d) HadCM3 historical simulation. 1 GJ = 1 × 10^9^ J.

HadCM3 and BZ2020 have similar patterns of $H_{e}$ changes during 1951–2008 (Figure 2 left column, changes are integrated over 0–2,000 m). In both of them, $H_{e}$ tends to accumulate in the subtropical gyres and the North Atlantic, but features little signal at low latitudes. HadCM3 has larger $H_{e}$ changes than BZ2020 in the North Atlantic and the Arctic. This is partly due to different definitions of $H_{e}$. The $H_{e}$ of BZ2020 is defined in a fixed‐circulation scenario, which has a smaller *Q*_ERF_ + *Q*′, hence a smaller $H_{e}$, than a free‐circulation scenario (i.e., the HadCM3 simulation) at northern high latitudes (Winton et al., 2013). $H_{r}$ is less coherent in space than $H_{e}$ in both HadCM3 and BZ2020; the two data sets show very different $H_{r}$ changes in the subpolar North Atlantic and the Southern Ocean (Figure 2, right column).

### 2.4 Heat Uptake by Control Ocean Transport

How important is the control ocean transport *L*_ctrl_ in shaping the regional pattern of $H_{e}$? We investigate this question using the pseudo excess temperature $\Theta_{e}^{*}$

(8) $$\begin{array}{ll} & \frac{\partial \Theta_{e}^{*}}{\partial t}+L_{\text{ctrl}}\left(\Theta_{e}^{*}\right)=\frac{1}{\rho_{0}c_{p}dz_{1}}\left(Q_{\text{ERF}}+Q^{\prime }\right),\\ & \text{initial condition:}\Theta_{e}^{*}\left(0\right)=0. \end{array}$$

$\Theta_{e}^{*}$ is evolved by the control transport *L*_ctrl_, as if heat uptake does not affect ocean transports. This is in contrast with Θ_e_ which is evolved by the historical transport *L*_hist_. We implement Equation 8 by simulating $\Theta_{e}^{*}$ as a passive tracer in the control experiment from 1860 to 2008. Pseudo excess heat content is denoted as $H_{e}^{*}=\int_{V}\rho_{0}c_{p}\Theta_{e}^{*}d^{3}r$.

**Figure 3:**
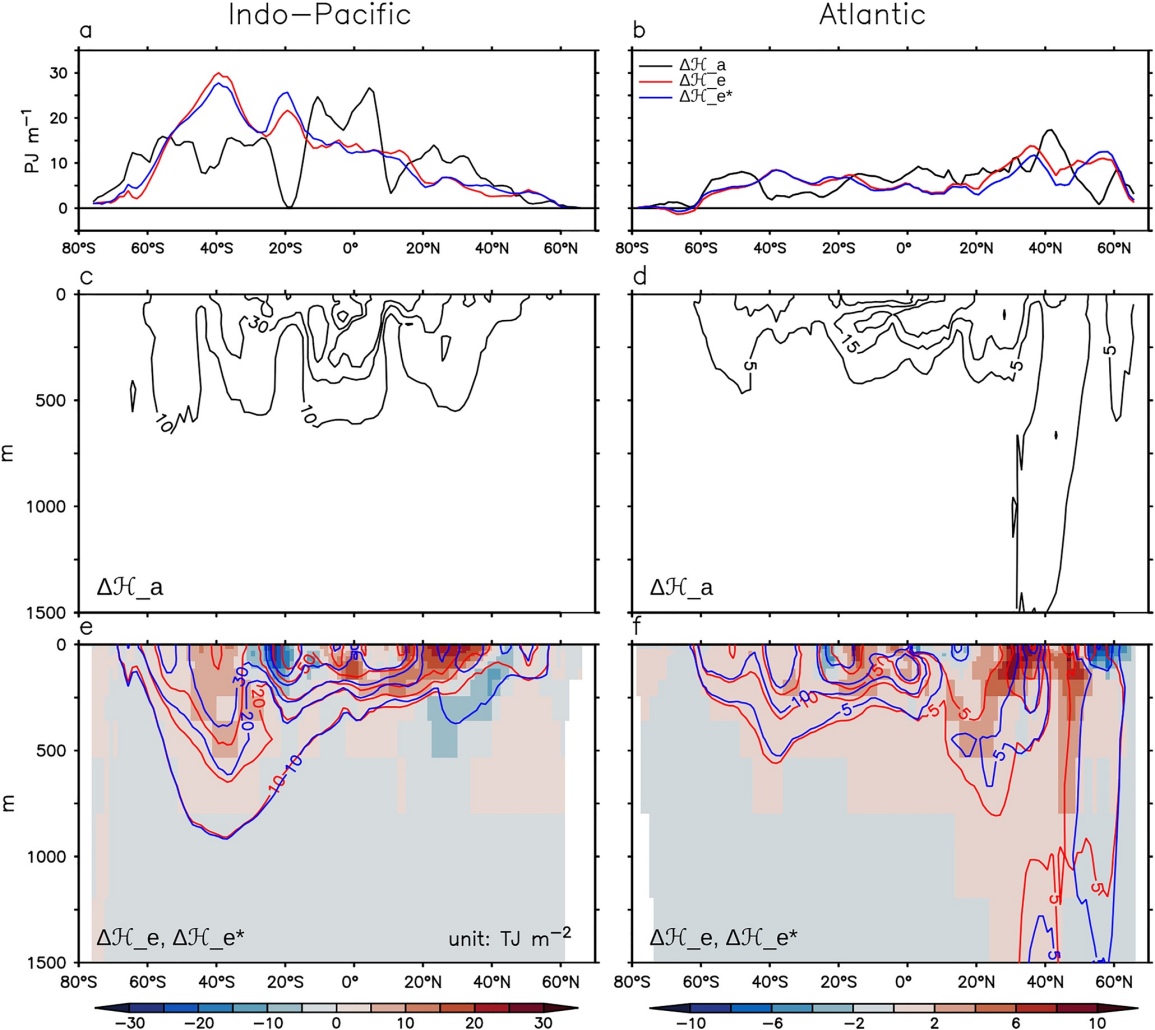
Excess heat content change resulting from (1) historical and (2) control ocean transports. These two quantities are denoted as $\Delta H_{e}$ (red line) and $\Delta H_{e}^{*}$ (blue line), respectively. Total heat content change ($\Delta H_{a}$ = $\Delta H_{e}$+$\Delta H_{r}$) is shown as black lines. (a and b) Zonal‐and‐depth integrated change (0–2,000 m). (c and f) Depth distribution of panels (a and b). A change is calculated as the difference between 1999–2008 and 1946–1955. In panels (e and f), contours indicate $\Delta H_{e}$ and $\Delta H_{e}^{*}$; shading indicates $\Delta H_{e}$ minus $\Delta H_{e}^{*}$. Contour levels are 10, 30, and 45 in panel (c); 5, 15, and 25 in panel (d); 10, 20, 30, 50, and 70 in panel (e); and 5, 10, 15, and 25 in panel (f). 1 PJ = 1 × 10^15^ J. 1 TJ = 1 × 10^12^ J.

Changes of $H_{a}$, $H_{e}$ and $H_{e}^{*}$ in 1999–2008 relative 1946–1955 are shown in Figure 3 (a change is denoted as “Δ”). $\Delta H_{a}$ is much less uniform than $\Delta H_{e}$ and $\Delta H_{e}^{*}$ across latitudes (Figures 3a and 3b), highlighting the role of $\Delta H_{r}$ in shaping the patterns of $\Delta H_{a}$. A similar result is found in Zika et al. (2021) but on a shorter timescale (2006–2017).

The latitude distributions of $\Delta H_{e}$ and $\Delta H_{e}^{*}$ are very similar, especially in the southern subtropics (Figure 3, compare red and blue lines). This suggests that the patterns of $\Delta H_{e}$ is mostly driven by the climatological ocean transport (i.e., *L*_ctrl_), not its transient response (i.e., differences between *L*_hist_ and *L*_ctrl_). A similar conclusion was found in several climate models under 1% increase of the atmospheric CO_2_ concentration (Couldrey et al., 2021; Gregory et al., 2016). Differences between $\Delta H_{e}$ and $\Delta H_{e}^{*}$ are most evident at northern mid latitudes, where $\Delta H_{e}$ is redistributed equatorward relative to $\Delta H_{e}^{*}$ in 0–200 m (Figures 3e and 3f shading). This redistribution pattern implies a weakening of the poleward ocean transport in the historical simulation.

## 3 Formulating Tracer Evolution Using Green's Functions

Evolution of a passive tracer in the historical simulation is governed by

(9) $$\begin{array}{ll} & \left(\frac{\partial }{\partial t}+L_{\text{hist}}\right)X\left(r,t\right)=\Psi \left(r,t\right),\\ & \Psi \left(r,t\right)=0\;\text{for}\;r\in \left\{\text{ocean interior}\right\}. \end{array}$$

**r** is a 3D position vector of the ocean and *t* is time. *X* is the concentration of a passive tracer and Ψ is its 3D source/sink. All tracers studied here have no source/sink in the ocean interior, therefore we set Ψ to zero everywhere below the surface. At the surface, $\Psi =F/dz_{1}$, where F is air‐sea tracer fluxes, and d*z*_1_ the top layer thickness. (F has a unit of K m s^−1^ for Θ_e_.)

### 3.1 Concentration GF Formulation

The general solution of Equation 9 is given in Holzer and Hall (2000). When *X* has zero initial conditions, the solution of Equation 9 can be written as a superposition of all tracer pulses emitted from the surface. For concentration BCs, the superposition is given as

(10) $$X\left(r,t\right)=\int_{\Omega }d^{2}r_{s}\int_{-\infty }^{t}G_{c}\left(r,t|r_{s},t_{s}\right)X^{s}\left(r_{s},t_{s}\right)dt_{s}.$$

*X* ^s^ is *X* at the surface. **r**_s_ is a surface position vector. *G*_c_ is the GF of Equation 9 that propagates concentration BCs. Ω denotes the global ocean surface. The two integrals sum up *X*(**r**, *t*) emitted from *X* ^s^ anywhere in Ω and anytime prior to *t*.

Formally *G*_c_ is defined as a special solution of Equation 9 that satisfies

(11) $$\begin{array}{ll} & \left(\frac{\partial }{\partial t}+L_{\text{hist}}\right)G_{c}\left(r,t|r_{s},t_{s}\right)=0,\\ & G_{c}\left(r,t|r_{s},t_{s}\right)=\delta \left(r-r_{s}\right)\delta \left(t-t_{s}\right)\;\text{for}\;r\in \Omega ,\\ & G_{c}\left(r,0|r_{s},t_{s}\right)=0\;\text{for}\;r\in \left\{\text{ocean interior}\right\}. \end{array}$$

*δ* is the Dirac delta function.

### 3.2 Interpretations of Concentration GFs

*G*_c_ can be interpreted from two perspectives. When we fix the surface coordinate (**r**_s_, *t*_s_), *G*_c_(**r**, *t*) is a time‐evolving 3D field in the ocean. The 3D field depicts how a tracer injected at (**r**_s_, *t*_s_) spreads in the ocean subject to zero concentration BCs at all other times and surface locations. The BCs remove any tracer that surfaces after *t*_s_, hence we have $\underset{\tau \to \infty }{\lim}G_{c}=0$, where *τ* = *t* − *t*_s_ is elapsed time. This perspective is useful for probing GFs from forward simulations in an ocean model (see Section 4).

When we fix the field coordinate (**r**, *t*), *G*_c_(**r**_s_, *t*_s_) is a time‐evolving 2D map of the ocean surface. It shows how sensitive *X*(**r**, *t*) is to individual pulses in its surface history *X* ^s^. Holzer and Hall (2000) interpreted the 2D map as a measure of how a tracer injected at (**r**, *t*) surfaces in the time‐reversed flow after *t* − *t*_s_. This perspective is useful for inferring GFs from tracer data (see Section 6). Causality requires that *G*_c_ = 0 whenever *t* <*t*_s_.

*G*_c_(**r**, *t* | **r**_s_, *t*_s_) is also referred to as a “joint water‐mass composition and transit‐time distribution” which measures the fraction of water at (**r**, *t*) that has made its last surface contact at location **r**_s_ and time *t*_s_ (Haine & Hall, 2002). Since all the water at (**r**, *t*) can be traced back to the surface eventually, the following must be satisfied.

(12) $$\int_{\Omega }d^{2}r_{s}\int_{-\infty }^{t}G_{c}\left(r,t|r_{s},t_{s}\right)dt_{s}=1.$$

*G*_c_ has been used to study the transit‐time distribution of the ocean (e.g., Ito & Wang, 2017; Maltrud et al., 2010; Peacock & Maltrud, 2006) and to estimate the ocean's uptake of anthropogenic carbon and heat (Gebbie & Huybers, 2019; Khatiwala et al., 2009; Newsom et al., 2020; Zanna et al., 2019).

### 3.3 Air‐Sea Flux GF Formulation

The solution of Equation 9 can also be written in terms of the air‐sea tracer flux $F_{X}$, when *X* has zero initial conditions.

(13) $$X\left(r,t\right)=\int_{\Omega }d^{2}r_{s}\int_{-\infty }^{t}G_{f}\left(r,t|r_{s},t_{s}\right)F_{X}\left(r_{s},t_{s}\right)dt_{s}$$

*G*_f_ is the boundary GF that propagates the surface source/sink into the ocean. Formally *G*_f_ is defined as a special solution of Equation 9 that satisfies

(14) $$\begin{array}{ll} & \left(\frac{\partial }{\partial t}+L_{\text{hist}}\right)G_{f}\left(r,t|r_{s},t_{s}\right)=\frac{1}{dz_{1}}\delta \left(r-r_{s}\right)\delta \left(t-t_{s}\right),\\ & G_{f}\left(r,0|r_{s},t_{s}\right)=0\;\text{every where}. \end{array}$$

Note that Equation 14 does not impose zero concentration BCs as Equation 11, hence tracers are not removed when they surface. *G*_f_ has been used to probe atmosphere transports (Holzer, 1999) and to define a tracer age (Holzer & Hall, 2000).

### 3.4 Limitation of Boundary GFs

We want to stress that *G*_c_ and *G*_f_ are both *boundary* GFs; that is they only account for tracers emitted from the surface. The redistributed temperature Θ_r_ cannot be accounted for using *G*_c_ or *G*_f_ because it has non‐zero source below the surface (Equation 7).

## 4 Simulating GFs in an Ocean Model

### 4.1 Approximations of Simulated GFs

The boundary GFs, *G*_c_ and *G*_f_, can be generated for an ocean model by simulating passive tracers in it. By definition, we need to compute a GF for every possible (**r**_s_, *t*_s_), which is computationally demanding. To reduce computational cost, we make the following approximations.

First, we assume that ocean transports are constant. Taking *G*_c_ as an example, this assumption means: (a) *G*_c_ is the same for *L*_ctrl_ and *L*_hist_, (b) *G*_c_ does not depend on *t*_s_, hence *G*_c_(**r**, *t* | **r**_s_, *t*_s_) = *G*_c_(**r**, *t* − *t*_s_ | **r**_s_, 0). Note that *G*_c_(**r**, *t* − *t*_s_ | **r**_s_, 0) only needs to be solved once (at *t*_s_ = 0) for every **r**_s_. The constant‐transport assumption neglects variability and forced‐change in ocean transports; we refer to the resulting errors as a “unforced‐transport error” and a “forced‐transport error,” respectively.

**Figure 4:**
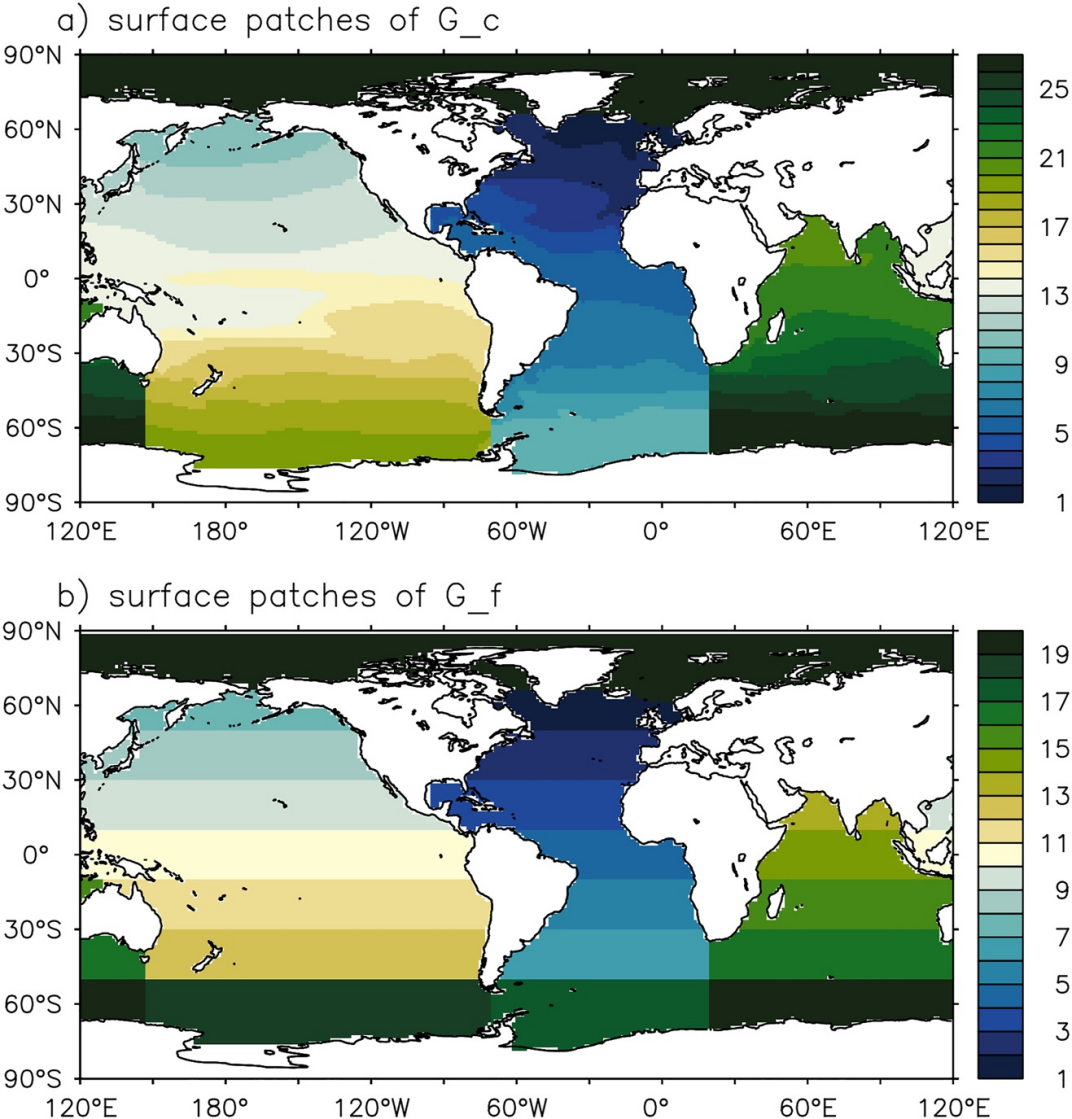
Surface patches for simulating boundary Green's functions *G*_c_ and *G*_f_. Shading indicates the patch index. *G*_c_ propagates concentration boundary conditions, while *G*_f_ propagates surface sources/sinks.

Second, we assume that the boundary terms, *X* ^s^ and $F_{X}$, are dominated by large‐scale patterns, hence we can approximate tracers emitted from them using coarse‐grained GFs. Specifically, we derive GFs using surface patches defined in Figure 4. For *G*_c_, we divide the global ocean into 27 regions based on the climatological surface densities in HadCM3, similar to Khatiwala et al. (2009). For *G*_f_, we divide the global ocean into 20° latitude bands for each basin (20 patches in total). This step greatly reduces the dimension of GFs at the surface (the dimension of **r**_s_ is about 1 × 10^4^ in a 1° × 1° model). *X* ^s^ and $F_{X}$ are averaged onto the corresponding patches when convolved with GFs.

Finally, we approximate the Dirac delta function in Equations 11 and 14 using a boxcar (rectangular) function with a unit height. The boxcar function lasts for 1 year after it is activated, so that the resulting GFs capture the effect of ocean transports averaged over a year, not that of a particular month. Using surface patches and the boxcar function neglects the covariance between GFs and the boundary terms within patches and years. We refer to this error as a “patch error.”

### 4.2 Defining Simulated GFs

GFs resulting from the above approximations are referred to as simulated GFs. Formally simulated concentration GF *G*_c_ (denoted as ${\hat{G}}_{c}$) is defined as

(15) $$\begin{array}{ll} & \left(\frac{\partial }{\partial t}+L_{\text{ctrl}}\right){\hat{G}}_{c}\left(r,t|p,0\right)=0,\\ & {\hat{G}}_{c}\left(r,t|p,0\right)=M_{c}\left(r,p\right)M_{t}\left(t,0\right)\text{for}\;r\in \Omega ,\\ & {\hat{G}}_{c}\left(r,0|p,0\right)=0\;\text{for}\;r\in \left\{\text{ocean interior}\right\}. \end{array}$$

The boxcar function *M*_*t*_(*t*, 0) is activated when *t* > 0 and switched off after 1 year. *t* = 0 is assigned to the beginning of the control experiment. *M*_c_ is a mask function that returns unity if **r** is within the surface patch *p* and zero otherwise. Ω denotes the global ocean surface. Similarly, simulated air‐sea flux GF *G*_f_ (denoted as ${\hat{G}}_{f}$) is defined as

(16) $$\begin{array}{ll} & \left(\frac{\partial }{\partial t}+L_{\text{ctrl}}\right){\hat{G}}_{f}\left(r,t|p,0\right)=\frac{1}{dz_{1}}M_{f}\left(r,p\right)M_{t}\left(t,0\right),\\ & {\hat{G}}_{f}\left(r,0|p,0\right)=0\;\text{every where}. \end{array}$$

*M*_f_ is a different mask function than *M*_c_, because we use different surface patches to define ${\hat{G}}_{f}$ and ${\hat{G}}_{c}$ (Figure 4). ${\hat{G}}_{c}$ and ${\hat{G}}_{f}$ are solved by integrating Equations 15 and 16, respectively, in the control experiment for 200 years (from 1860).

### 4.3 Estimating Tracers Using Simulated GFs

Simulated GFs can be used to compute the boundary response of a passive tracer following

(17) $$\hat{X}\left(r,l\right)=\sum_{p}\sum_{l_{s}\leq l}\left[{\hat{G}}_{c}\left(r,l-l_{s}|p,0\right)\right]{\hat{X}}^{s}\left(p,l_{s}\right),$$

(18) $$\hat{X}\left(r,l\right)=\sum_{p}\sum_{l_{s}\leq l}\left[{\hat{G}}_{f}\left(r,l-l_{s}|p,0\right)\right]{\hat{F}}_{X}\left(p,l_{s}\right).$$

All quantities in Equations 17 and 18 are defined on a yearly grid. ${\hat{X}}^{s}$ and ${\hat{F}}_{X}$ are *X* ^s^ and $F_{X}$ averaged onto the corresponding patches, respectively. *p* is surface patch index and *l* is year number. To reduce the unforced‐transport error, we simulate GFs from four initial conditions (1860, 1870, 1880, and 1890 of the control experiment), and use their ensemble means (denoted by “[ ]”) in Equations 17 and 18.

We introduce $\sum \hat{G}{\hat{\chi }}^{s}$ as a shorthand for $\sum_{p}\sum_{l_{s}\leq l}\hat{G}\left(r,l-l_{s}|p,0\right){\hat{\chi }}^{s}\left(p,l_{s}\right)$. Using this notation, the ${\hat{G}}_{c}$ estimate of Θ_e_ can be written as ${\hat{\Theta }}_{e}=\sum \left[{\hat{G}}_{c}\right]{\hat{\Theta }}_{e}^{s}$ (substitute Θ_e_ for *X* in Equation 17), where ${\hat{\Theta }}_{e}^{s}$ is Θ_e_ at the surface averaged onto patches. Similarly, the ${\hat{G}}_{c}$ estimate of $\Theta_{e}^{*}$ can be written as ${\hat{\Theta }}_{e}^{*}=\sum \left[{\hat{G}}_{c}\right]{\hat{\Theta }}_{e}^{s*}$.

### 4.4 Error Definitions

${\hat{\Theta }}_{e}$ is inaccurate because of the patch, unforced‐transport and forced‐transport errors. We quantify these errors using Equations (19), (20), (21). Because $\Theta_{e}^{*}$ is not affected by forced change in ocean transports, we use it to isolate the patch and unforced‐transport errors. We note that different members of ${\hat{G}}_{c}$ give similar estimates of $\Theta_{e}^{*}$ for large‐scale changes examined in Section 5; the spread is <20% compared to the ensemble mean in most regions (not shown). Similarly, Maltrud et al. (2010) suggested that the first moment of ${\hat{G}}_{c}$ (i.e., mean age) can be robustly estimated using just a few members. We therefore assume that the ${\hat{G}}_{c}$ estimate of $\Theta_{e}^{*}$, that is $\sum \left[{\hat{G}}_{c}\right]{\hat{\Theta }}_{e}^{s*}$, is dominated by the patch error (Equation 19, $\Theta_{e}^{*}$ without “^” is the model truth). The change of the ${\hat{G}}_{c}$ estimate resulting from replacing $\left[{\hat{G}}_{c}\right]$ with ${\hat{G}}_{c}$ (a single realization) gives the unforced‐transport error (Equation 20). A larger ensemble of ${\hat{G}}_{c}$ is useful to refine the unforced‐transport error; however, it is unlikely to change the error substantially (e.g., by a factor of two).

(19) $$patch\text{\_}error=\sum \left[{\hat{G}}_{c}\right]{\hat{\Theta }}_{e}^{s*}-\Theta_{e}^{*}$$

(20) $$unforced\text{\_}transport\text{\_}error=\sum {\hat{G}}_{c}{\hat{\Theta }}_{e}^{s*}-\sum \left[{\hat{G}}_{c}\right]{\hat{\Theta }}_{e}^{s*}$$

Because Θ_e_ is evolved by the historical ocean transport, we use it to compute the forced‐transport error. The ${\hat{G}}_{c}$ estimate of Θ_e_, that is $\sum \left[{\hat{G}}_{c}\right]{\hat{\Theta }}_{e}^{s}$, is affected by the patch and forced‐transport error; the patch error is solved in Equation 19, therefore the forced‐transport error is given as Equation 21.

(21) $$forced\text{\_}transport\text{\_}error=\sum \left[{\hat{G}}_{c}\right]{\hat{\Theta }}_{e}^{s}-\Theta_{e}-patch\text{\_}error$$

In practice, there is also a “BC error” when estimating Θ_e_ using ${\hat{G}}_{c}$. This is because surface excess temperature $\left(\Theta_{e}^{s}\right)$ is poorly known. Using SST anomaly $\left(\Theta_{a}^{s}\right)$ to approximate $\Theta_{e}^{s}$ is not accurate because $\Theta_{a}^{s}$ is contaminated by redistributed temperature. We compute the BC error by replacing $\Theta_{e}^{s}$ with $\Theta_{a}^{s}$ in the ${\hat{G}}_{c}$ estimate (Equation 22).

(22) $$BC\text{\_}error=\sum \left[{\hat{G}}_{c}\right]{\hat{\Theta }}_{a}^{s}-\sum \left[{\hat{G}}_{c}\right]{\hat{\Theta }}_{e}^{s}.$$

We compute the errors for the ${\hat{G}}_{f}$ estimate (Equation 18) in a similar way as for the ${\hat{G}}_{c}$ estimate (Equation 17). The only difference is that the boundary term $F_{X}$ is the same for $\Theta_{e}^{*}$ and Θ_e_ in the ${\hat{G}}_{f}$ estimate by definition.

### 4.5 Surface Concentration BCs

$\Theta_{e}^{s*}$, $\Theta_{e}^{s}$ and $\Theta_{a}^{s}$ are supplied as anomalies relative to 1860–1880 when evaluating Equation 17. This step is to exclude a shock in $\Theta_{e}^{s}$ (+0.15°C in global mean) shortly after the start of the historical simulation. Because $\Theta_{e}^{s*}$ does not show a similar behavior, we suspect that the shock is due to an abrupt change in ocean transports. If not removed, the shock would cause a warm bias in the ${\hat{G}}_{c}$ estimate of Θ_e_, because ${\hat{G}}_{c}$ is derived from the control experiment. Since this warm bias can be removed easily, we do not count it as a forced‐transport error.

**Figure 5:**
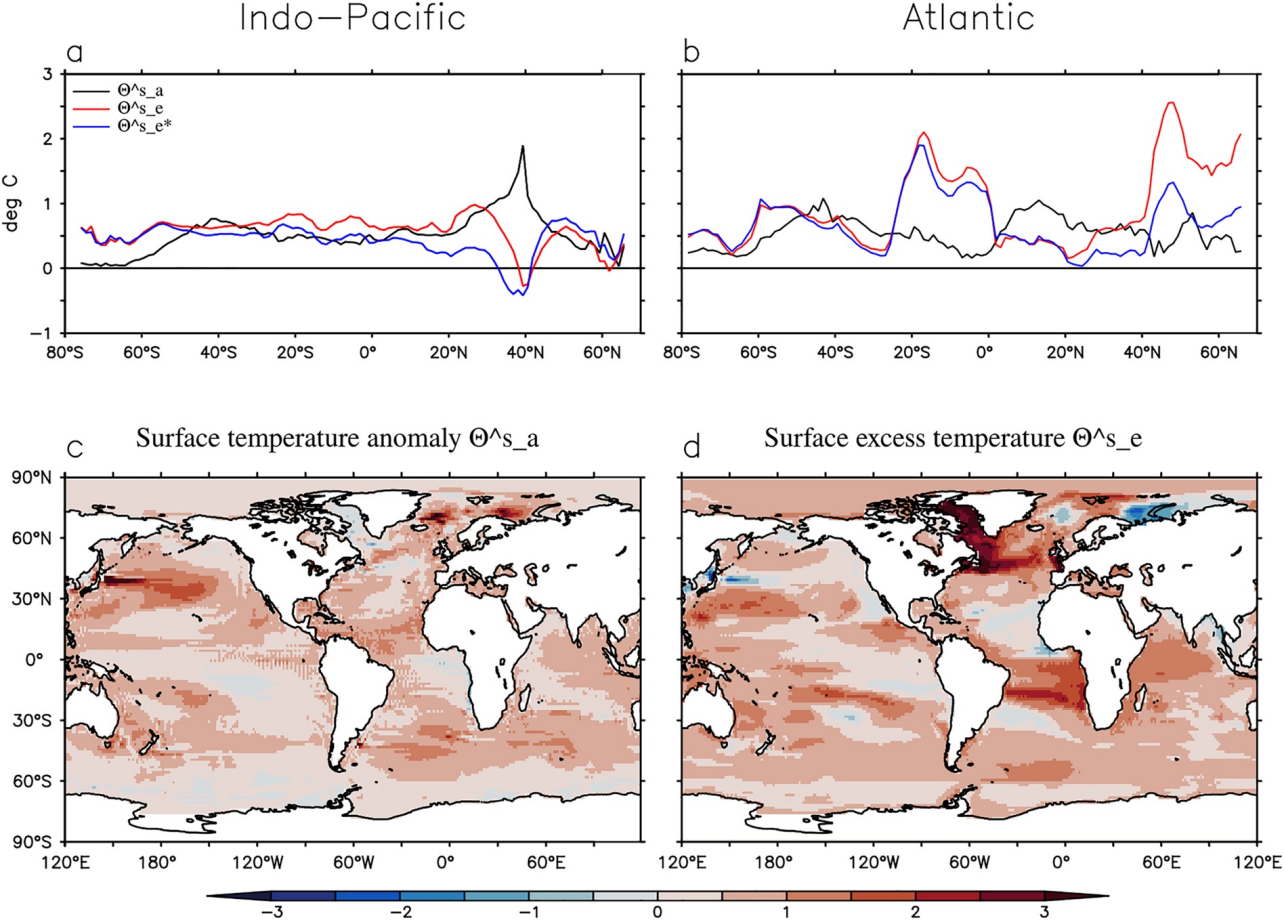
Surface temperature anomaly $\left(\Theta_{a}^{s}\right)$ and surface excess temperature ($\Theta_{e}^{s}$ and $\Theta_{e}^{s*}$). $\Theta_{e}^{s*}$ is the same as $\Theta_{e}^{s}$ except that it is evolved by the control ocean transport instead of the historical one. Values shown are differences between 1999–2008 and 1860–1880. (a and b) Zonal average over a basin. (c and d) Spatial map.

A comparison between $\Theta_{a}^{s}$, $\Theta_{e}^{s}$ and $\Theta_{e}^{s*}$ is shown in Figure 5 for the 1999–2008 average. $\Theta_{a}^{s}$ (black line) consistently has less warming than $\Theta_{e}^{s}$ (red line) in the Southern Ocean and the North Atlantic (north of 40°N) by as much as 2°C (Figures 5a and 5b). This difference is likely caused by a reduction of convection and a slow down of the Atlantic meridional overturning circulation, as shown in Gregory et al. (2016).

$\Theta_{e}^{s}$ (red line) and $\Theta_{e}^{s*}$ (blue line) are very similar at most latitudes (Figures 5a and 5b). The exception is the North Pacific and the North Atlantic, where $\Theta_{e}^{s}$ is much warmer than $\Theta_{e}^{s*}$. This implies a reduction of the ocean's surface‐to‐interior transport in those regions during the historical simulation (because global warming stratifies the ocean and thus inhibits heat uptake).

### 4.6 Potential Nonlinear Errors

Equation 19 assumes that the function Φ is strictly linear when operating on passive tracers in models. This is not necessarily true because some models use flux‐limited transport schemes, which makes Φ nonlinear even when operating on passive tracers. This nonlinear error is included in the error computed from Equation 19, but it is likely small compared to the patch error.

Excess temperature Θ_e_ can alternatively be defined as a dynamical tracer that affects ocean transports (i.e., replacing *L*_hist_ with Φ in Equation 6). This definition leads to a set of “dynamical” GFs, as opposed to “passive” GFs of Section 3 (their distinctions are further discussed in Appendix B). The GF estimate of the dynamical Θ_e_ (Equation B1) contains a nonlinear error because the dynamical ocean response is not a linear function of the forcing. In contrast, the GF estimate of the passive Θ_e_ (Equation 6) does not have a nonlinear error, because Equation 6 is strictly linear. The errors introduced in Section 4.1 can all be eliminated by simulating GFs for Equation 6 at very fine space and time resolution. The nonlinear error, however, cannot be eliminated by any means.

## 5 Estimating Excess Heat Using Simulated GFs

In this section, we examine how well simulated GFs can reproduce excess heat changes in the historical simulation. The inaccuracy is partitioned into the patch, unforced‐transport, forced‐transport and BC errors (Equations (19), (20), (21), (22)).

**Figure 6:**
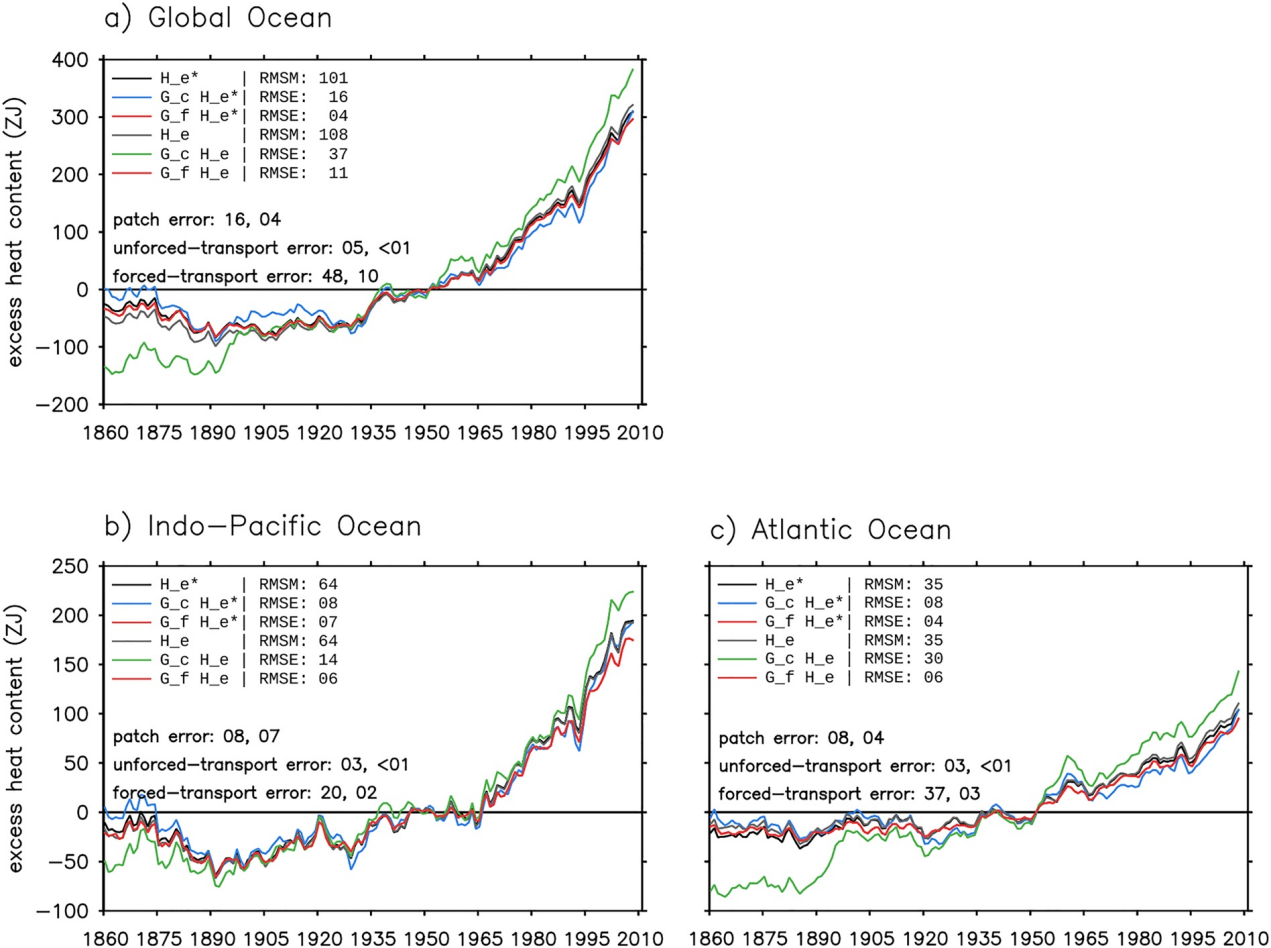
Estimating the global/basin integrated (0–2,000 m) excess heat $H_{e}$ and $H_{e}^{*}$ in the historical simulation using simulated Green's functions (GFs) (Sections 5.1 and 5.2). Black and gray lines show $H_{e}^{*}$ and $H_{e}$ in HadCM3, respectively. $H_{e}^{*}$ is the same as $H_{e}$ except that it is evolved by the control ocean transport. Blue and green lines are the ${\hat{G}}_{c}$ estimates of $H_{e}^{*}$ and $H_{e}$, respectively. The ${\hat{G}}_{f}$ estimates of $H_{e}^{*}$ and $H_{e}$ are identical and both shown by red lines. The root‐mean‐square magnitude (RMSM) of the model truth and the root‐mean‐square errors (RMSEs) of different GF estimates are listed. The RMS values of the patch, unforced‐transport and forced‐transport errors are listed for the ${\hat{G}}_{c}$ and ${\hat{G}}_{f}$ estimates from left to right. The two ${\hat{G}}_{f}$ estimates have different RMSEs because $H_{e}^{*}$ and $H_{e}$ are different in the model truth.

**Figure 7:**
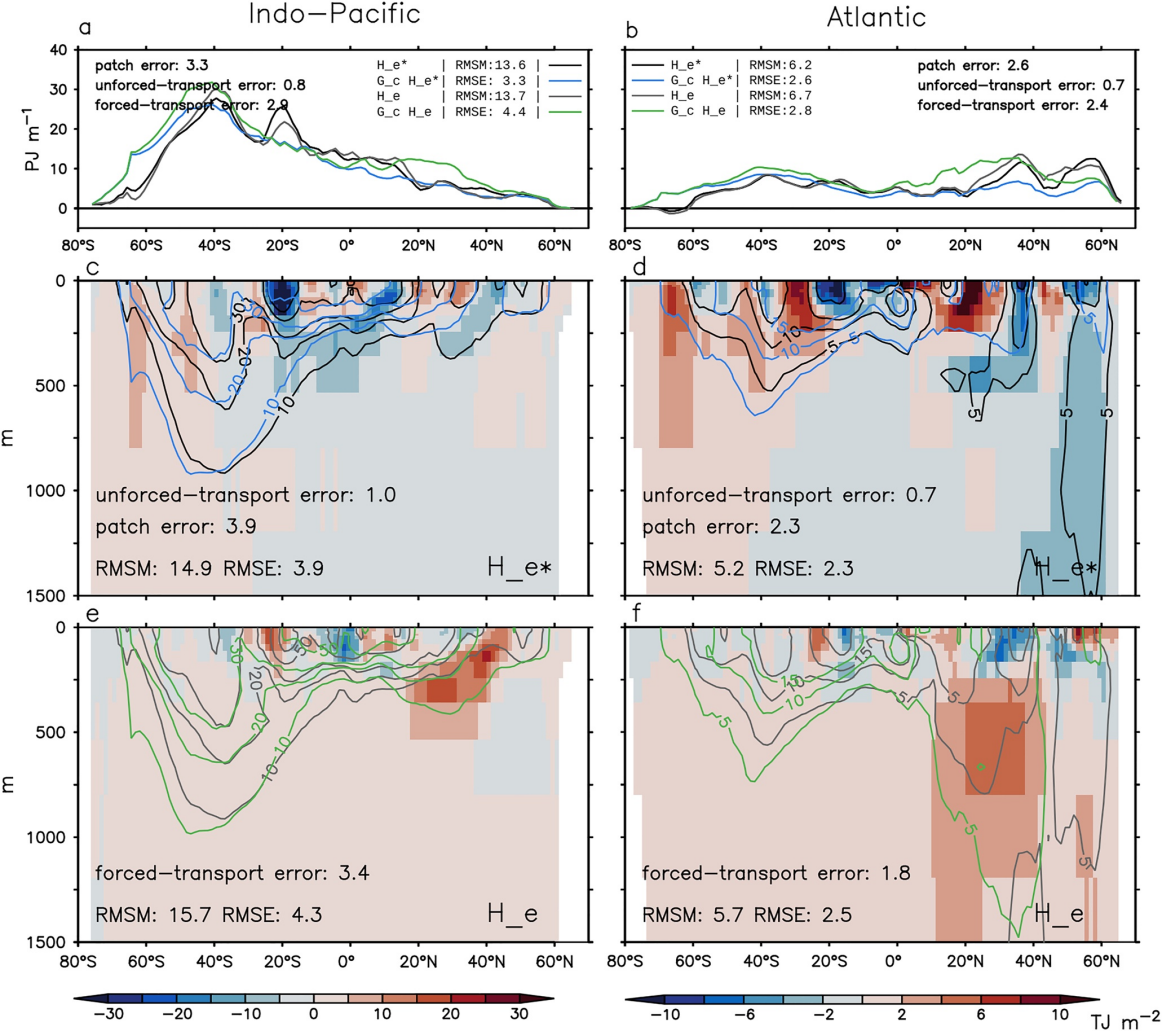
Estimating latitude distribution of excess heat change $\Delta H_{e}$ and $\Delta H_{e}^{*}$ in the historical simulation using ${\hat{G}}_{c}$ (Section 5.1). (a and b) Zonal‐and‐depth integral (0–2,000 m). (c–f) Depth distribution of panels (a and b). In all panels, black and gray lines show $\Delta H_{e}^{*}$ and $\Delta H_{e}$ in HadCM3, respectively; blue and green lines show the ${\hat{G}}_{c}$ estimates of $\Delta H_{e}^{*}$ and $\Delta H_{e}$, respectively. Shading in panels (c and d) indicates errors in the ${\hat{G}}_{c}$ estimate of $\Delta H_{e}^{*}$ (the patch error). Shading in panels (e and f) indicates errors in the ${\hat{G}}_{c}$ estimate of $\Delta H_{e}$ minus the patch error (the forced‐transport error). For each metric, the root‐mean‐square magnitude (RMSM) of the model truth and the root‐mean‐square error (RMSE) of the ${\hat{G}}_{c}$ estimate are listed, along with the RMS values of the patch, unforced‐transport and forced‐transport errors. All changes are calculated as differences between 1999–2008 and 1946–1955.

**Figure 8:**
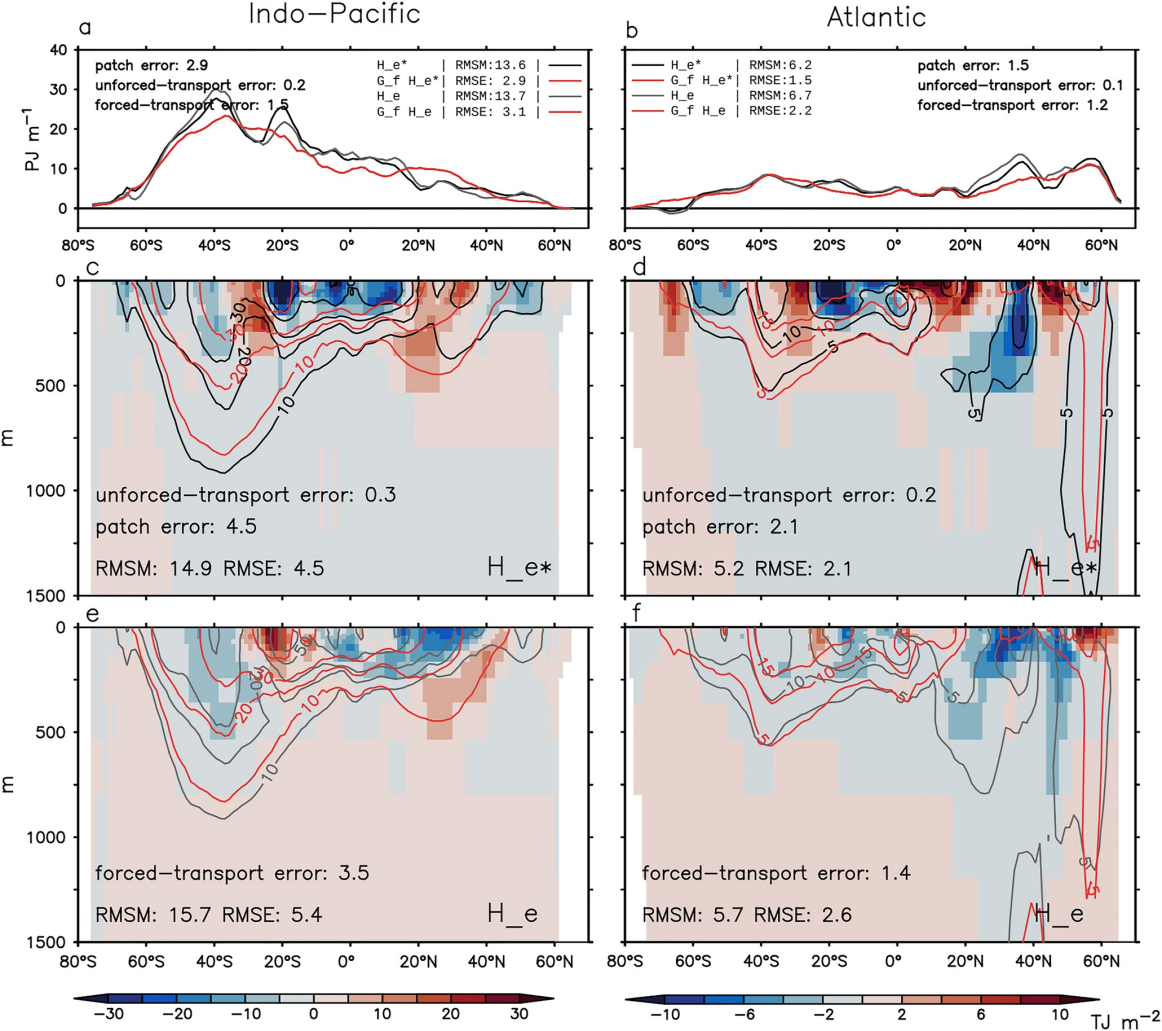
Estimating latitude distribution of excess heat change $\Delta H_{e}$ and $\Delta H_{e}^{*}$ in the historical simulation using ${\hat{G}}_{f}$ (Section 5.2). (a and b) Zonal‐and‐depth integral (0–2,000 m). (c–f) Depth distribution of panels (a and b). In all panels, black and gray lines show $\Delta H_{e}^{*}$ and $\Delta H_{e}$ in HadCM3, respectively; the ${\hat{G}}_{f}$ estimates of $\Delta H_{e}^{*}$ and $\Delta H_{e}$ are identical, and shown by red lines. Shading in panels (c and d) indicates errors in the ${\hat{G}}_{f}$ estimate of $\Delta H_{e}^{*}$ (the patch error). Shading in panels (e and f) indicates errors in the ${\hat{G}}_{f}$ estimate of $\Delta H_{e}$ minus the patch error (the forced‐transport error). For each metric, the root‐mean‐square magnitude (RMSM) of the model truth and the root‐mean‐square error (RMSE) of the ${\hat{G}}_{f}$ estimate are listed, along with the RMS values of the patch, unforced‐transport and forced‐transport errors. The two ${\hat{G}}_{f}$ estimates have different RMSEs because $H_{e}^{*}$ and $H_{e}$ are different in the model truth. All changes are calculated as differences between 1999–2008 and 1946–1955.

We focus on three metrics when comparing the model truth with the GF estimates: (a) global/basin volume integral (0–2,000 m), (b) zonal‐and‐depth integral (0–2,000 m), and (c) depth distribution of (b) (0–1500 m). Metric (a) is shown as anomalies relative to 1946–1955. Metrics (b) and (c) are shown as changes between 1999–2008 and 1946–1955 (denoted using “Δ”). The root‐mean‐square error (RMSE) of the GF estimate (total error) and the RMS value for each error source (Equations (19), (20), (21), (22)) are listed in Figures 6, 7, 8. Each realization of GFs gives an unforced‐transport error; we report the unforced‐transport error averaged over four realizations. Since our metrics are all extensive quantities, we also report their normalized RMSEs; that is the ratio between RMSE and root‐mean‐square magnitude (RMSM).

### 5.1 Concentration GF Estimate

In this subsection, we evaluate the GF estimate of $H_{e}$ and $H_{e}^{*}$ based on Equation 17 (referred to as the ${\hat{G}}_{c}$ estimate). $H_{e}^{*}$ is the same as $H_{e}$ except that it is evolved by the control ocean transport (see Section 2.4). ${\hat{G}}_{c}$ is simulated in the control experiment. The BCs $\Theta_{e}^{s}$ and $\Theta_{e}^{s*}$ are diagnosed in HadCM3 (i.e., BCs are perfectly known). Θ_e_ and $\Theta_{e}^{*}$ are converted to excess heat $H_{e}$ and $H_{e}^{*}$, respectively, following the procedure of Section 2.2.4. We exclude $H_{e}$ and $H_{e}^{*}$ resulting from the Arctic patch to be consistent with Zanna et al. (2019).

#### 5.1.1 Patch and Unforced‐Transport Errors

We start with $H_{e}^{*}$. The ${\hat{G}}_{c}$ estimate of $H_{e}^{*}$ is inaccurate because of the patch error. For all metrics, the RMS value of the unforced‐transport error is less than 1/3 of the patch error (compare numbers in Figures 6 and 7). The global integrated $H_{e}^{*}$ (black line) increases by about 300 ZJ over 1860–2008, of which two thirds are stored in the Indo‐Pacific and one third in the Atlantic (Figure 6). For this metric, the ${\hat{G}}_{c}$ estimate (blue line) reproduces the model truth well, with a RMSE of 16 ZJ for the global integral and 8 ZJ for basin integrals (Figure 6).

The zonal‐and‐depth integrated $\Delta H_{e}^{*}$ (black line) has a RMSM of 13.6 PJ m^−1^ in the Indo‐Pacific and 6.2 PJ m^−1^ in the Atlantic, averaged over latitudes (Figures 7a and 7b). The ${\hat{G}}_{c}$ estimate of this metric (blue line) has an error of 24% in the Indo‐Pacific and 42% in the Atlantic (Figures 7a and 7b). The patch error is most evident in the North Atlantic (underestimate) and in the Southern Ocean (overestimate) (Figures 7a and 7b, compare black and blue lines).

The latitude‐depth pattern of $\Delta H_{e}^{*}$ (black contour) has a RMSM of 14.9 TJ m^−2^ in the Indo‐Pacific and 5.2 TJ m^−2^ in the Atlantic, averaged over latitudes and 0–1,500 m (Figures 7c and 7d). The ${\hat{G}}_{c}$ estimate of this metric (blue contour) has an error of 26% in the Indo‐Pacific and 44% in the Atlantic (Figures 7c and 7d). The patch error is strongest in the upper 200 m (Figures 7c and 7d shading). Below that, the ${\hat{G}}_{c}$ estimate follows the model truth broadly, except in the Atlantic around 60°N (Figures 7c and 7d, compare black and blue contours).

#### 5.1.2 Forced‐Transport Error

We next examine the ${\hat{G}}_{c}$ estimate of $H_{e}$. By definition, this estimate is inaccurate because of the patch and forced‐transport errors; we subtract the patch error (Section 5.1.1) from the total error to derive the forced‐transport error (Equation 21). Global warming stratifies the ocean and weakens the surface‐to‐interior transport. The forced‐transport error arises because ${\hat{G}}_{c}$ does not capture this weakening effect, hence tends to overestimate warming at depths. This is evident in Figure 6; the ${\hat{G}}_{c}$ estimate (green line) overestimates $H_{e}$ (gray line) in both global and basin integrals. The overestimate is strongest at northern mid latitudes (Figures 7e and 7f shading).

In the North Atlantic, the forced‐transport error is associated with a 1 Sv slowdown of the overturning circulation at 45°N after 1960 (not shown). In contrast, the overturning circulation shows little change compared to the control in the North Pacific, implying the forced‐transport error there is associated with parameterized transports. Interestingly, the forced‐transport error is nearly zero in the Southern Ocean. This is probably because the Southern Ocean circulation has a strong wind‐driven component (Marshall & Speer, 2012), hence is less sensitive to surface warming compared to the North Atlantic circulation.

The forced‐transport error is more than twice as large as the patch error for global/basin integrated $H_{e}$ (Figure 6), and about the same size as the patch error for zonal integrated $\Delta H_{e}$ (Figure 7). The patch and forced‐transport errors partially compensate each other for zonal integrated $\Delta H_{e}$ in some regions (Figure 7, compare middle and bottom row shading), hence the RMSE of the ${\hat{G}}_{c}H_{e}$ estimate is only slightly larger than that of the ${\hat{G}}_{c}H_{e}^{*}$ estimate in Figure 7.

### 5.2 Air‐Sea Flux GF Estimate

In this subsection, we evaluate the GF estimates of $H_{e}$ and $H_{e}^{*}$ based on Equation 18 (referred to as the ${\hat{G}}_{f}$ estimate). ${\hat{G}}_{f}$ is simulated in the control experiment. The BCs $F_{\Theta_{e}}$ are diagnosed in HadCM3 as (*Q*_ERF_ + *Q*′)/(*ρ*_0_ *c*_*p*_). $F_{\Theta_{e}^{*}}$ is the same as $F_{\Theta_{e}}$ by definition, therefore the ${\hat{G}}_{f}$ estimates of $H_{e}$ and $H_{e}^{*}$ are identical. Θ_e_ and $\Theta_{e}^{*}$ are converted to excess heat $H_{e}$ and $H_{e}^{*}$, respectively, following the procedure of Section 2.2.4.

The ${\hat{G}}_{f}$ estimate has smaller RMSEs than the ${\hat{G}}_{c}$ estimate for global/basin integrals (Figure 6) and zonal‐and‐depth integrals (Figures 7 and 8, top row). In particular, the unforced‐ and forced‐transport errors of the ${\hat{G}}_{f}$ estimate are much smaller than those of the ${\hat{G}}_{c}$ estimate. Note that the basin integrated $H_{e}^{*}$ and $H_{e}$ are largely determined by their surface fluxes, which are directly supplied to the ${\hat{G}}_{f}$ estimate.

In contrast, the ${\hat{G}}_{f}$ estimate is less accurate than the ${\hat{G}}_{c}$ estimate for the latitude‐depth patterns of $\Delta H_{e}^{*}$ and $\Delta H_{e}$ in the Indo‐Pacific (compare Figure 7 with Figure 8 for (c) and (e)). This is because the ${\hat{G}}_{f}$ estimate has a larger patch error, especially in 0–200 m (compare shading in Figure 7c with Figure 8c). In the Atlantic, the ${\hat{G}}_{f}$ and ${\hat{G}}_{c}$ estimates have similar RMSEs for the latitude‐depth patterns of $\Delta H_{e}^{*}$ and $\Delta H_{e}$, but the forced‐transport error is smaller in the ${\hat{G}}_{f}$ estimate, especially below 200 m (compare shading in Figure 7f with Figure 8f).

### 5.3 GF Estimate in a Real‐World Application

In this subsection, we simulate a real‐world application of the GF method in the model world. Specifically, we estimate excess heat $H_{e}$ in the historical simulation using: (a) simulated $\Theta_{a}^{s}$ and (b) ${\hat{G}}_{c}$ derived from HadCM3. This setup corresponds to Zanna et al. (2019) who reconstructed the real‐world $H_{e}$ by combining: (a) observed $\Theta_{a}^{s}$ and (b) ${\hat{G}}_{c}$ derived from an ocean model. To distinguish the $\Theta_{e}^{s}$‐based ${\hat{G}}_{c}$ estimate (examined in Section 5.1) from the $\Theta_{a}^{s}$‐based ${\hat{G}}_{c}$ estimate (to be examined below), we refer to the latter as the ${\hat{G}}_{c}^{+}$ estimate. The ${\hat{G}}_{c}^{+}$ estimate suffers an additional BC error compared to the ${\hat{G}}_{c}$ estimate, because of the differences between $\Theta_{e}^{s}$ and $\Theta_{a}^{s}$ (see Section 4.5).

#### 5.3.1 BC Error

**Figure 9:**
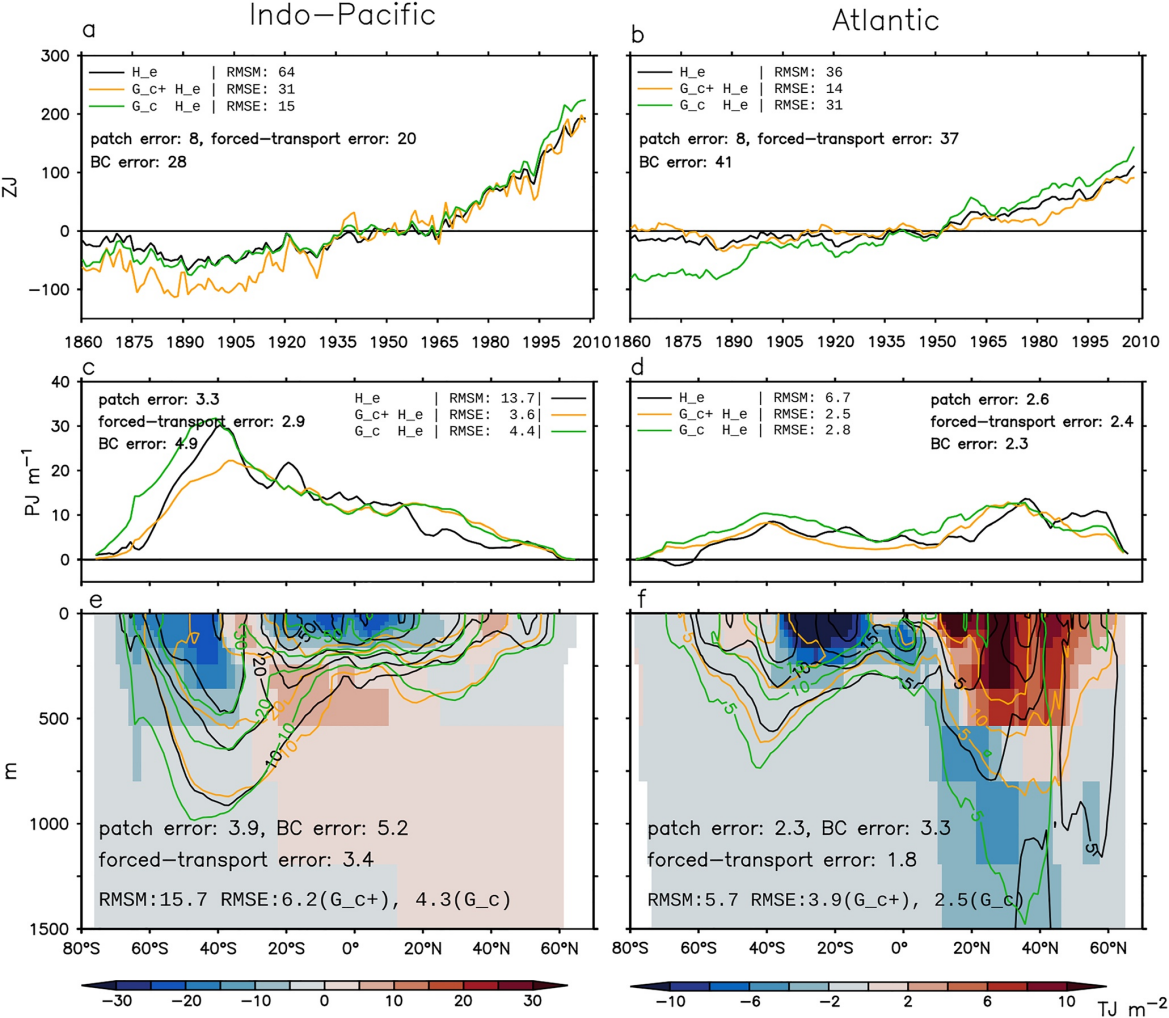
Estimating excess heat $H_{e}$ in the historical simulation using ${\hat{G}}_{c}$ and $\Theta_{a}^{s}$ (Section 5.3). This estimate is referred to as the ${\hat{G}}_{c}^{+}$ estimate. (a and b) Basin‐volume integral. (c and d) Zonal‐and‐depth integrated change (0–2,000 m). (e and f) Depth distribution of panels (c and d). In all panels, black lines are the model truth, orange lines are the ${\hat{G}}_{c}^{+}$ estimate, and green lines are the ${\hat{G}}_{c}$ estimate in Figures 6 and 7. Shading in panels (e and f) indicates the boundary condition (BC) error (Equation 22). For each metric, the root‐mean‐square magnitude (RMSM) of the model truth and the root‐mean‐square errors (RMSEs) of the two Green's function estimates are listed, along with the RMS values of the patch, forced‐change and BC errors.

The BC error is the largest error for the latitude‐depth pattern of $\Delta H_{e}$ (Figures 9e and 9f); it is as large as the forced‐transport error for basin integrated $H_{e}$ (Figures 9a and 9b) and depth integrated $\Delta H_{e}$ (Figures 9c and 9d). For zonal‐and‐depth integrated $\Delta H_{e}$, the BC error causes an underestimate in most of the Atlantic and south of 40°S of the Indo‐Pacific (Figures 9c and 9d, compare orange and green lines). In the Southern Ocean, the underestimate caused by the BC error partially compensates the overestimate caused by the patch and forced‐transport errors, reducing the total error there.

#### 5.3.2 Total Error

When all error terms are considered, the ${\hat{G}}_{c}^{+}$ estimate (orange line) reconstructs the model truth (black line) with an error of 48% and 39% for basin integrated $H_{e}$ in the Indo‐Pacific and Atlantic, respectively (Figures 9a and 9b). In the Indo‐Pacific, the total error is 26% for zonal‐and‐depth integrated $\Delta H_{e}$ and 39% for its depth distribution (Figures 9c and 9e). These numbers are larger in the Atlantic, at 37% and 68%, respectively (Figures 9d and 9f). For depth‐integrated $\Delta H_{e}$, the largest error occurs at mid and high latitudes, for example, an overestimate in the North Pacific (Figures 9c and 9d, compare black and orange lines).

## 6 Inferring GFs From Tracer Data

### 6.1 Introducing the Inverse Problem

The GF model Equation 10 connects *X* at (**r**, *t*) with its surface history *X* ^s^ via *G*_c_ at **r**. This forms a constraint on *G*_c_ at **r** for every pair of *X*(**r**, *t*) and *X* ^s^ in observations. In this section, we introduce a method to infer *G*_c_ from such constraints. This problem is the “inverse” of the forward problem discussed in Section 5, which uses *X* ^s^ and *G*_c_ at **r** to estimate *X*(**r**, *t*). Inferring *G*_c_ is useful as it only requires tracer data; for example, one can use it to estimate the real‐world *G*_c_ from observed tracers.

Observations are insufficient constraints on *G*_c_. We assume that ocean transports are constant to reduce the number of unknowns in *G*_c_. This allows us to rewrite *G*_c_(**r**, *t*, | **r**_s_, *t*_s_) as *G*_c_(**r**, 0, | **r**_s_, *t*_s_ − *t*) and rewrite Equation 10 as

(23) $$X_{n}\left(r,t_{n}\right)=\int_{\Omega }d^{2}r_{s}\int_{-\infty }^{t_{n}}G_{c}\left(r,0|r_{s},t_{s}-t_{n}\right)X_{n}^{s}\left(r_{s},t_{s}\right)dt_{s}.$$

*n* is an index for different observations; *t*_*n*_ is the time of the *n*th observation. For example, one can assign *n* = 1 to CFC‐11 observed at year 1994, *n* = 2 to CFC‐12 at year 2000 and *n* = 3 to CFC‐12 at year 1994. Note that *G*_c_ in Equation 23 depends on *t*_*s*_ − *t*_*n*_, not *t*_*n*_ alone, which is different from *G*_c_ in Equation 10. Causality requires that *G*_c_(**r**, 0 | **r**_s_, *τ*) = 0 for *τ* > 0, where *τ* = *t*_*s*_ − *t*_*n*_.

### 6.2 Maximum Entropy Method

At every **r**, *N* observations of *X*_*n*_ and $X_{n}^{s}$ impose *N* constraints on *G*_c_ via Equation 23. In practice, *N* is much smaller than the number of unknowns in *G*_c_; the latter is at least the number of locations in **r**_s_. Among infinitely many *G*_c_ that satisfy constraints, we choose the one that is the most “similar” to an initial guess of *G*_c_ (denoted as *G*_pr_). This method is called the Maximum Entropy (MaxEnt) method and was first applied to infer *G*_c_ by Khatiwala et al. (2009) and Holzer et al. (2010). Formally, the above procedure can be cast as a constrained optimization problem, and solved using the method of Lagrangian multipliers.

Given *N* observations of tracers at **r**, *X*_*n*_(**r**, *t*_*n*_), *n* = 1, …, *N*, the MaxEnt estimate of *G*_c_ is a function of $X_{n}^{s}$ and *G*_pr_

(24) $$G_{ME}\left(r,0|r_{s},\tau \right)=\frac{1}{Z}G_{pr}\left(r,0|r_{s},\tau \right)\exp\left(\sum_{n=1}^{N}a_{n}X_{n}^{s}\left(r_{s},t_{n}+\tau \right)\right).$$

*Z* is a normalization factor to ensure that *G*_ME_ integrates to unity over the global ocean surface (Ω) and all *τ* values, which is required by Equation 12. To determine the *N* unknowns (*a*_1_, …, *a*_*N*_), we substitute *G*_ME_ and $X_{n}^{s}$ into Equation 23

(25) $$X_{n}^{\prime }\left(r,t_{n}|a_{1},\cdots ,a_{N}\right)=\int_{\Omega }d^{2}r_{s}\int_{-\infty }^{t_{n}}G_{ME}\left(r,0|r_{s},t_{s}-t_{n}\right)X_{n}^{s}\left(r_{s},t_{s}\right)dt_{s},$$

and solve for *a*_1_, …, *a*_*N*_ that minimize the misfit between *X*_*n*_ and $X_{n}^{\prime }$. Formally, the desired *a*_*n*_ is given as

(26) $$\underset{a}{\mathrm{arg min}}\left(\|x^{\prime }\left(a\right)-x{\|}_{2}^{2}+\lambda \|a{\|}_{2}^{2}\right),$$

where **x**, **x**′ and **a** are column vectors of *X*_*n*_, $X_{n}^{\prime }$ and *a*_*n*_, respectively. ‖ ⋅ ‖ is the 2‐norm of a vector (i.e., $\|a{\|}_{2}^{2}=a_{1}^{2}+\cdots +a_{N}^{2}$). Each row of **x**′(**a**) and **x** are normalized so that model‐data misfits of different tracers are comparable. The regularization term $\lambda \|a{\|}_{2}^{2}$ is included to prevent overfitting, because there are errors in observations and in Equation 23. We set *λ* to unity based on the L‐curve method (Hansen & O’Leary, 1993). Derivation of Equation 24, the L‐curve method, and how we choose *λ* are described in Appendix C.

There are other methods to infer *G*_c_ from observations. For example, Gebbie and Huybers (2010) and DeVries and Primeau (2011) estimate the operator *L* (Equation 9) from observations. Once *L* is derived, one can use it to calculate *G*_c_ analytically.

### 6.3 Transient Tracers in the Ocean

#### 6.3.1 Introducing CFCs, SF_6_, and Bomb Δ^14^C

Observations of CFC‐11, CFC‐12, and SF_6_ (Fine, 2011) are often used as data constraints in the MaxEnt method (i.e., *X*_*n*_ in Equation 23). CFCs and SF_6_ are man‐made chemical tracers that have been released into the atmosphere since the 1930s and gradually taken up by the ocean. CFCs and SF_6_ are stable in the oxygenated open ocean. Once entering the ocean, they are advected and diffused by ocean transports, like passive tracers.

We also explore the use of bomb ^14^C as data constraints in the MaxEnt method. ^14^C is commonly expressed as Δ^14^C, which is the deviation of the ^14^C/^12^C ratio relative to a standard value. ^14^C is naturally generated in the atmosphere by cosmic rays. The ^14^C content of a water parcel decays with a half‐life of 5,730 years once it is out of contact with the atmosphere. During the 1950s and 1960s, the nuclear weapon tests dramatically increased Δ^14^C in the atmosphere. This “bomb Δ^14^C” signal invades the ocean in a way similar to CFCs and SF_6_.

#### 6.3.2 Spatial Distribution

**Figure 10:**
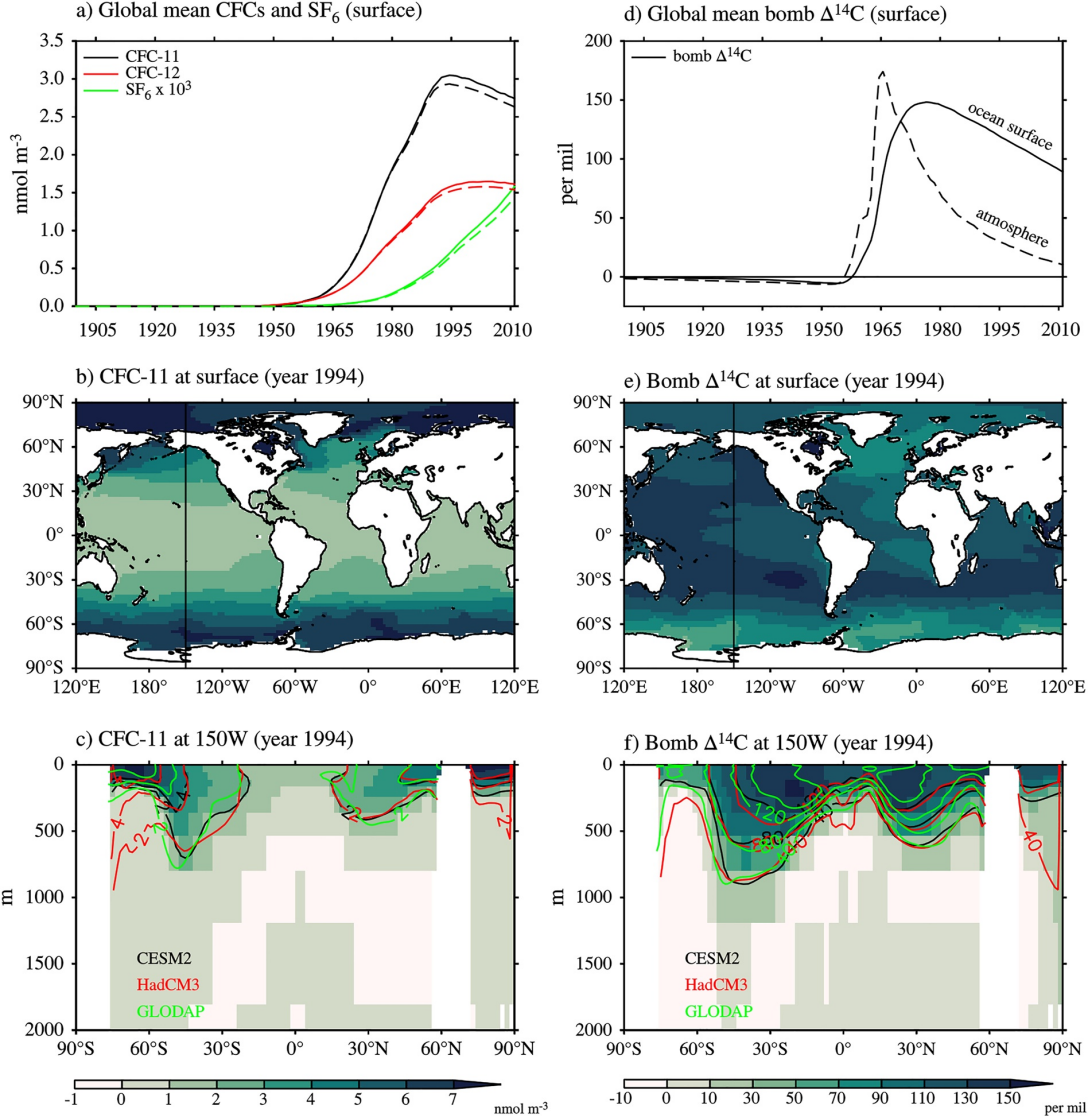
Transient tracers in the ocean from the CESM2 historical simulation. (a) Sea surface concentrations of CFC‐11, CFC‐12, and SF_6_ (solid lines) and their atmosphere mixing ratios (dashed lines). Both quantities are shown as global mean. Dashed lines are multiplied by arbitrary scaling factors. (b and c) CFC‐11 at year 1994 at the surface and the 150°W section (shading and black contours). For these two metrics, CFC‐12 and SF_6_ have similar patterns compared to CFC‐11, but with different magnitudes. Panels (d–f) are the same as panels (a–c), but for bomb Δ^14^C. CFC‐11 and bomb Δ^14^C in HadCM3 (Section 6.4) and observations are shown as red and green contours, respectively, in the bottom row. 1 nmol = 1 × 10^−9^ mol.

We use results from a historical simulation of CESM2 (Danabasoglu et al., 2020) to demonstrate passages of CFCs, SF_6_ and bomb Δ^14^C in the ocean (Figure 10). The CESM2 historical simulation is conducted under the CMIP6 protocol (Eyring et al., 2016; Orr et al., 2017). We derive bomb Δ^14^C as anomalies in Δ^14^C relative to its 1850–1870 climatology. Measurements of CFCs, SF_6_ and Δ^14^C from historical cruises are made available as gridded and profile data by Global Ocean Data Analysis Project (GLODAP; Key et al., 2004; Olsen et al., 2016).

Both CFC‐11 and bomb Δ^14^C invade the ocean from the surface, similar to how excess heat is carried to depths, for example, at the 150°W section (Figures 10c and 10f, shading). A major difference between CFC‐11 and ^14^C is that the latter has a much longer air‐sea equilibration timescale than the former (10 years vs. weeks) (Broecker & Peng, 1974). This has two consequences for CFC‐11 and bomb Δ^14^C in the ocean. First, the surface CFC‐11 (solid line) follows its atmospheric history (dashed line) closely for global mean, while the surface bomb Δ^14^C shows a slower increase and decay compared to its atmospheric history (Figures 10a and 10d). Second, and more importantly, the surface CFC‐11 and bomb Δ^14^C have very different patterns in the ocean (compare Figures 10b and 10e), because the pattern of bomb Δ^14^C is more affected by ocean transports (due to slow air‐sea equilibration). CFC‐12 and SF_6_ have similar air‐sea equilibration timescales and spatial patterns as CFC‐11.

### 6.4 Simulated Tracer Observations

To derive *G*_ME_ as one would do with observations, we include CFCs, SF_6_, and bomb Δ^14^C in the historical simulation (see Appendix D for details). The resulting CFC‐11 and bomb Δ^14^C are similar to the gridded GLODAPv1 observations. Taking the 150°W section as an example, this is evident as a good agreement between red and green contours in Figures 10c and 10f. In polar regions, tracers in HadCM3 tend to penetrate to greater depths than those in CESM2, implying that HadCM3 has a stronger convection than CESM2 there. To isolate the forced‐transport error, we also simulate CFCs, SF_6_, and bomb Δ^14^C in the control experiment. In Section 7, we use simulated observations in the historical and control experiments to estimate $H_{e}$ and $H_{e}^{*}$, respectively.

### 6.5 A Baseline Setup for Computing *G*_ME_

It is important to note that *G*_ME_ is not uniquely defined, but depends on the choice of data constraints and priors. Because we want to test the application of *G*_ME_ in the real world, we construct a *G*_ME_ using HadCM3 equivalents of real‐world observations. We refer to this *G*_ME_ as *G*_MEb_.

#### 6.5.1 Data Constraints

We use four tracers simulated in HadCM3 to compute *G*_MEb_; they are CFC‐11 and CFC‐12 at year 1994 and climatological temperature and salinity. These four tracers are available in observations from the gridded GLODAPv1 data (Key et al., 2004). We choose this data set because it has a nearly global coverage, hence one could compute *G*_ME_ everywhere in the ocean.

For climatological temperature and salinity, we repeat their surface BCs in time, and truncate the time integral in Equation 25 from (−*∞*, *t*_*n*_] to [*t*_*n*_ − 7,999, *t*_*n*_] years. The 8,000‐year limit is an upper bound of the timescale to tracer equilibrium in the global ocean under concentration BCs. One can set *t*_*n*_ to an arbitrary number for climatological tracers because their *X*_*n*_ and $X_{n}^{s}$ are both constant in time.

#### 6.5.2 Space and Time Average

All data on the HadCM3 grid are averaged onto a 10° × 10° grid before solving for *G*_ME_. Because every interior point has a *G*_ME_, the spatial averaging reduces the total number of *G*_ME_ to be solved. Despite the low resolution, the coarse grid can still capture most of spatial variability in the surface BCs of CFCs, SF_6_ and bomb Δ^14^C, because they all exhibit coherent spatial structures (Figures 10b and 10e). On the time dimension, we focus on annually averaged quantities as for simulated GFs (Sections 4 and 5). After the coarse‐grained averaging, *G*_ME_(**r**, 0 | **r**_s_, *τ*) now becomes a 10° × 10° resolution 2D map defined on a yearly grid for a given **r**.

#### 6.5.3 Computing Prior GFs

We use ${\hat{G}}_{c}$ simulated in a 1,000‐year control run of the FAMOUS climate model as *G*_pr_ for *G*_MEb_.

(27) $$G_{pr}\left(r,0|r_{s},\tau \right)=\frac{1}{A\left(p\left(r_{s}\right)\right)}{\hat{G}}_{c}\left(r,-\tau |p\left(r_{s}\right),0\right),0\geq \tau \geq -999\;\text{years},$$

where *p*(**r**_s_) returns the index of surface patch that **r**_s_ is in and *A*(⋅) returns the surface area of a patch given its index. FAMOUS is a low resolution version of HadCM3, which uses most of the HadCM3 codes, but runs about 10 times faster (R. S. Smith et al., 2008). The FAMOUS ${\hat{G}}_{c}$ is generated using the same procedure and surface patches as described in Section 4. Because deep oceans ventilate on millennial timescales, *G*_pr_ derived from Equation 27 does not satisfy Equation 12 at every **r**. We fill in the rest of *G*_pr_ by assuming that the fraction of water ventilated at *τ* ≤ −1,000 years is uniformly distributed over a 7,000‐year period and the global ocean surface

(28) $$G_{pr}\left(r,0|r_{s},\tau \right)=\frac{1}{7,000A_{o}}\left(1-\sum_{\tau =0}^{999}\int_{\Omega }G_{pr}\left(r,0|r_{s},\tau \right)d^{2}r_{s}\right),-1,000\geq \tau \geq -7,999\;\text{years}.$$

*A*_0_ is the surface area of the global ocean $\left(A_{0}=\sum_{i=1}^{27}A\left(i\right)\right)$. We simply set *G*_pr_ to zero for *τ* ≤ −8,000 years.

## 7 Estimating Excess Heat Using Inferred GFs

In this section, we examine how well inferred GFs *G*_ME_ can reproduce excess heat change in the historical simulation. We derive *G*_ME_ by updating a prior estimate of *G*_c_ to fit simulated tracer observations (Section 6.2).

### 7.1 Error Definitions

The *G*_ME_ estimate of $H_{e}$ is inaccurate for the following reasons. First, the *G*_MEb_ estimate suffers an “information error,” because observations are insufficient constraints on *G*_c_. Although this problem is regularized by the MaxEnt method, it is likely that *G*_ME_ still differs from the true *G*_c_ in many aspects. The *G*_ME_ estimate also suffers the patch, unforced‐transport and forced‐transport errors like the ${\hat{G}}_{c}$ estimate discussed in Section 5. We assume that the former two error sources are small for the *G*_ME_ estimate because: (a) the *G*_ME_ estimate resolves surface BCs at 10° × 10° resolution; and (b) the unforced‐transport error is found small for the ${\hat{G}}_{c}$ estimate. We partition errors in the *G*_ME_ estimate into the information, forced‐transport and BC errors as below, similar to Section 4.4.

(29) $$information\text{\_}error=\sum G_{ME}\Theta_{e}^{s*}-\Theta_{e}^{*}$$

(30) $$forced\text{\_}transport\text{\_}error=\sum G_{ME}\Theta_{e}^{s}-\Theta_{e}-information\text{\_}error$$

(31) $$BC\text{\_}error=\sum G_{ME}\Theta_{a}^{s}-\sum G_{ME}\Theta_{e}^{s}$$

We compare the model truth with the *G*_ME_ estimates using the same metrics as Section 5. They are: (a) global/basin volume integral (0–2,000 m), (b) zonal‐and‐depth integral (0–2,000 m), and (c) depth distribution of (b) (0–1,500 m). All metrics are showed as anomalies relative to the 1946–1955 average. A change (denoted using “Δ”) is calculated as the difference between 1999–2008 and 1946–1955.

### 7.2 Evaluating a Baseline Estimate

In this subsection, we evaluate the *G*_MEb_ estimate of $H_{e}$ and $H_{e}^{*}$. This estimate is calculated from Equation 23, wherein we replace *G*_c_ with *G*_MEb_. We use *G*_MEb_ derived from the historical and control experiments to estimate $H_{e}$ and $H_{e}^{*}$, respectively. $H_{e}^{*}$ is the same as $H_{e}$ except that it is evolved by the control ocean transport (see Section 2.4). *G*_MEb_ is a particular *G*_ME_ constrained by simulated observations in HadCM3 (see Section 6.5). The BCs $\Theta_{e}^{s}$ and $\Theta_{e}^{s*}$ are diagnosed in HadCM3 (i.e., BCs are perfectly known). Note that Section 5 uses the same ${\hat{G}}_{c}$ to estimate $H_{e}$ and $H_{e}^{*}$, which is different from here.

#### 7.2.1 Information Error

**Figure 11:**
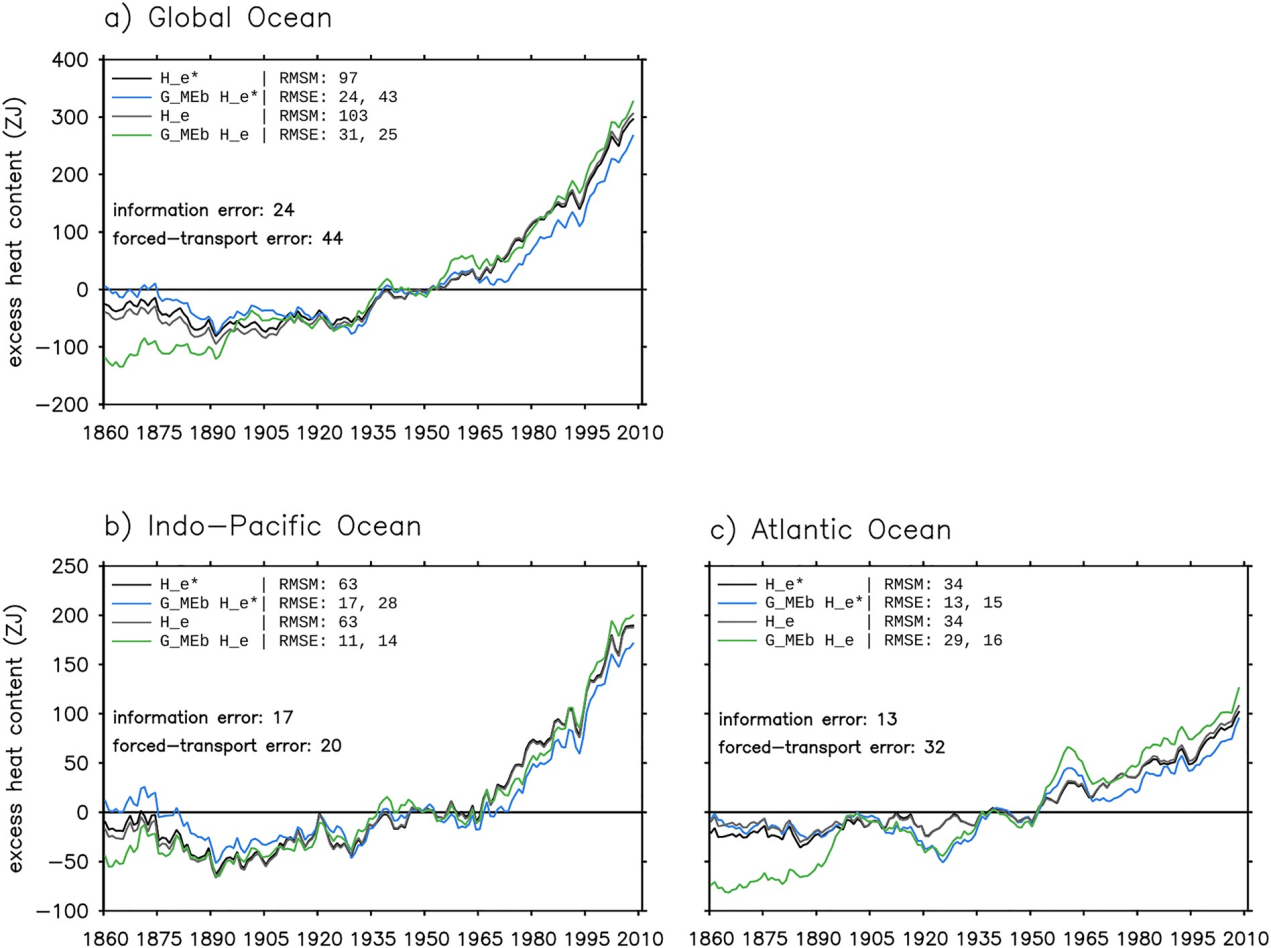
Estimating global/basin integrated (0–2,000 m) excess heat $H_{e}$ and $H_{e}^{*}$ in the historical simulation using *G*_MEb_ (Section 7.2). Black and gray lines show $H_{e}^{*}$ and $H_{e}$ in HadCM3, respectively. Blue and green lines are the *G*_MEb_ estimates of $H_{e}^{*}$ and $H_{e}$, respectively. $H_{e}^{*}$ is the same as $H_{e}$ except that it is evolved by the control ocean transport. The root‐mean‐square magnitude (RMSM) of the model truth, the root‐mean‐square errors (RMSEs) of the *G*_MEb_ estimate (first number) and the prior estimate (second number), and the RMS values of the information and forced‐transport errors are listed.

The *G*_MEb_ estimate (blue line) reproduces the global/basin integrated $H_{e}^{*}$ in HadCM3 (black line) well (Figure 11), with an error of 25% for the global ocean, 27% for the Indo‐Pacific and 38% for the Atlantic. (A percentage error is calculated as the ratio between RMSE and RMSM.) A constant 50 ZJ offset between the *G*_MEb_ estimate and the model truth is evident in Figure 11a after 1965 (compare blue and black lines).

**Figure 12:**
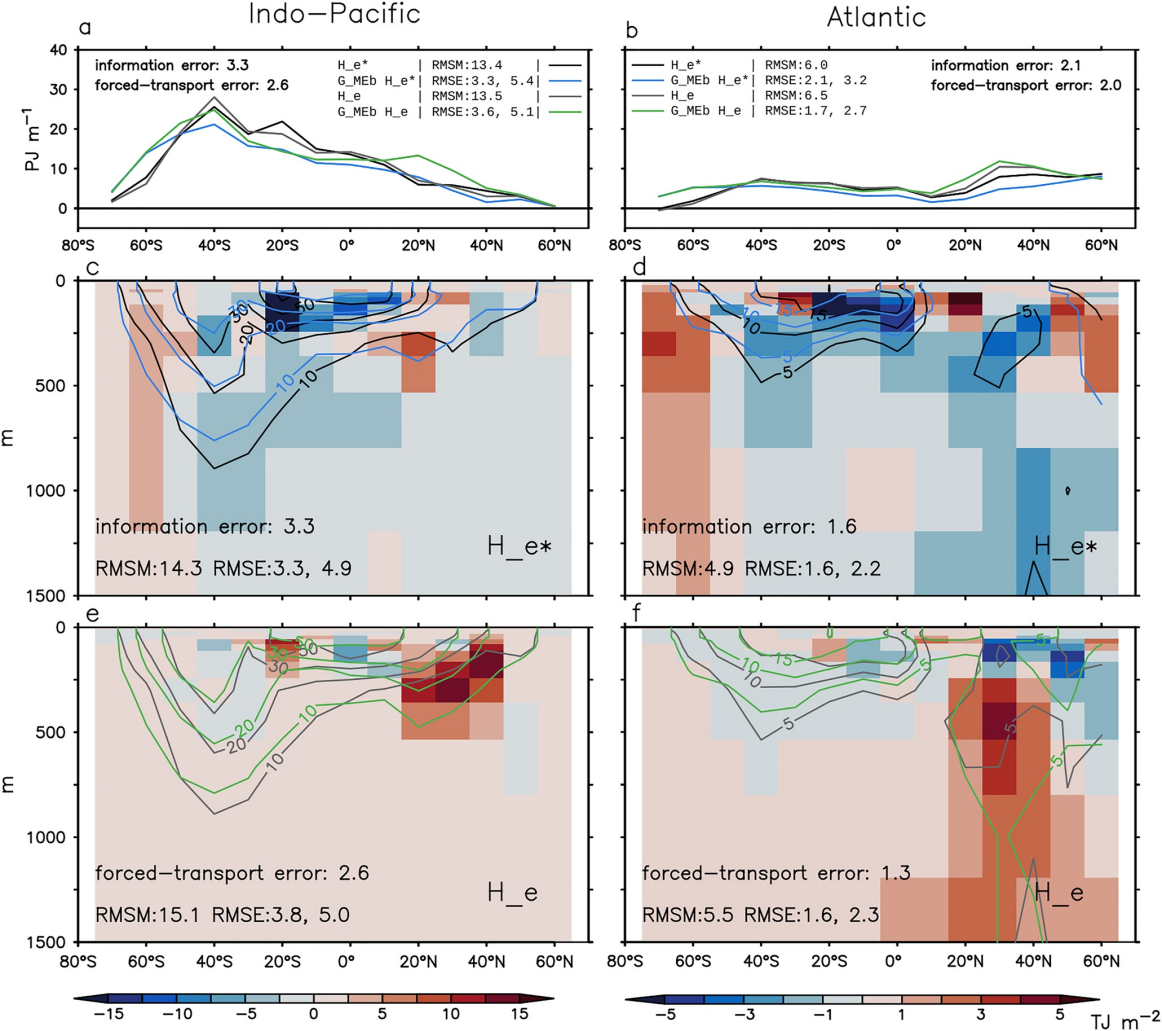
Estimating latitude distribution of excess heat change $\Delta H_{e}$ and $\Delta H_{e}^{*}$ in the historical simulation using *G*_MEb_ (Section 7.2). (a and b) Zonal‐and‐depth integral (0–2,000 m). (c–f) Depth distribution of panels (a and b). In all panels, black and gray lines show $H_{e}^{*}$ and $H_{e}$ in HadCM3, respectively; blue and green lines show the *G*_MEb_ estimates of $H_{e}^{*}$ and $H_{e}$, respectively. Shading in panels (c and d) indicates errors in the *G*_MEb_ estimate of $H_{e}^{*}$ (the information error). Shading in panels (e and f) indicates errors in the *G*_MEb_ estimate of $H_{e}$ minus the information error (the forced‐transport error). For each metric, the root‐mean‐square magnitude (RMSM) of the model truth, the root‐mean‐square errors (RMSEs) of the *G*_MEb_ estimate (first number) and the prior estimate (second number), and the RMS values of the information and forced‐transport errors are listed. All changes are calculated as differences between 1999–2008 and 1946–1955.

The *G*_MEb_ estimate broadly captures the latitude‐depth pattern of $\Delta H_{e}^{*}$ in the Indo‐Pacific and the Atlantic, with a greater error in the latter (Figures 12a–12d compare black and blue lines/contours). The error for depth integrated $\Delta H_{e}^{*}$ is 25% and 35% in the Indo‐Pacific and the Atlantic, respectively. In both basins, $\Delta H_{e}^{*}$ is underestimated by 0–5 PJ m^−1^ at most latitudes, except south of 50°S where it is overestimated (Figures 12a and 12b compare black and blue lines). The overestimate is evident over the 0–1,500 m depths, while the underestimate mostly comes from the 0–400 m depths (Figures 12c and 12d shading). For these zonal integrated metrics, the *G*_MEb_ estimate has a similar accuracy compared to the ${\hat{G}}_{c}$ estimate (Section 5.1) in both basins.

#### 7.2.2 Forced‐Transport Error

The forced‐transport error causes an overestimate in the *G*_MEb_ estimate, especially at northern mid latitudes (Figures 12e and 12f, shading), similar to that in the ${\hat{G}}_{c}$ estimate. The forced‐transport error is more than twice as large as the information error for the global and the Atlantic integrated $H_{e}$ (Figure 11), while it is about the same size as the information error for zonal integrated $\Delta H_{e}$ (Figure 12). In the Atlantic, the underestimate caused by the information error is partially compensated by the overestimate caused by the forced‐transport error, reducing the total error there (except south of 50°S) (Figure 12b).

#### 7.2.3 Effects of Data Constraints

How do data constraints improve on the initial guess *G*_pr_? We examine this question by comparing RMSEs between the *G*_MEb_ estimate and the *G*_pr_ estimate. The *G*_pr_ estimate is calculated using the same equation as the *G*_MEb_ estimate, except replacing *G*_MEb_ with *G*_pr_. The *G*_MEb_ estimate has a smaller RMSE compared to the *G*_pr_ estimate for all the metrics examined in Figures 11 and 12 (shown by numbers in the legends). The reduction of RMSE is between 20% and 40% (the number is different for different metrics). The exceptions are the global and the Atlantic integrated $H_{e}$, for which the *G*_MEb_ estimate has a greater RMSE than the *G*_pr_ estimate (Figure 11c). We suspect that this increase of RMSE is related to the forced‐transport error, because the same behavior is not found for $H_{e}^{*}$.

### 7.3 GF Estimate in a Real‐World Application

**Figure 13:**
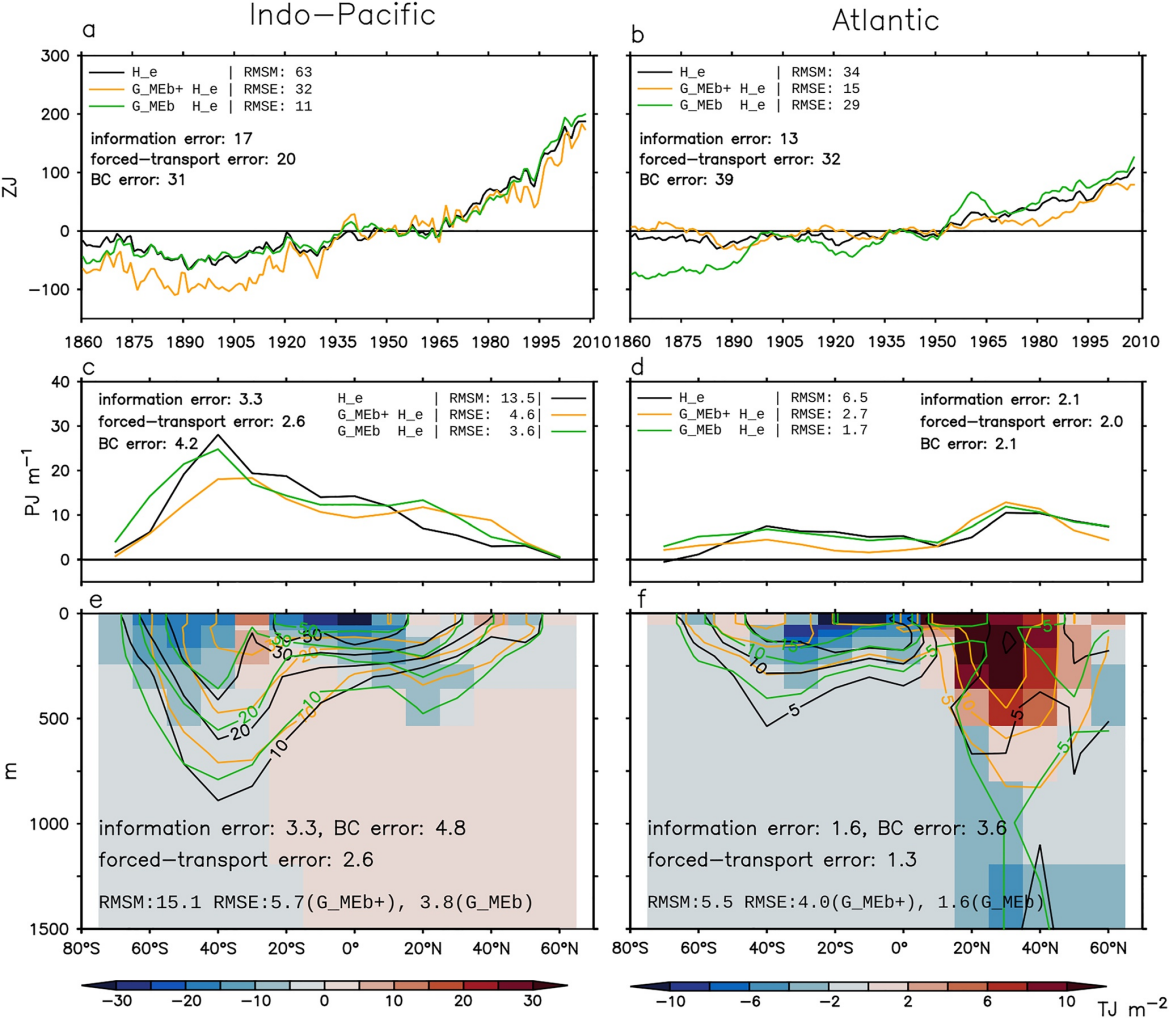
Estimating excess heat $H_{e}$ in the historical simulation using *G*_MEb_ and $\Theta_{a}^{s}$ (Section 7.3). This estimate is referred to as the $G_{MEb}^{+}$ estimate. (a and b) Basin‐volume integral. (c and d) Zonal‐and‐depth integrated change (0–2,000 m). (e and f) Depth distribution of panels (c and d). In all panels, black lines are the model truth, orange lines are the $G_{MEb}^{+}$ estimate, and green lines are the *G*_MEb_ estimate in Figures 11 and 12. Shading in panels (e–f) indicates the boundary condition (BC) error (Equation 31). For each metric, the root‐mean‐square magnitude (RMSM) of the model truth and the root‐mean‐square errors (RMSEs) of the two Green's function estimates are listed, along with the RMS values of the information, forced‐change and BC errors.

In this subsection, we simulate a real‐world application of the GF method in the model world. Specifically, we estimate excess heat $H_{e}$ in the historical simulation using: (a) simulated $\Theta_{a}^{s}$ and (b) *G*_MEb_ derived from simulated observations. This calculation can be repeated using the real‐world $\Theta_{a}^{s}$ and observations. To distinguish the $\Theta_{e}^{s}$‐based *G*_MEb_ estimate (examined in Sections 7.2) from the $\Theta_{a}^{s}$‐based *G*_MEb_ estimate (to be examined below), we refer to the latter as the $G_{MEb}^{+}$ estimate. The $G_{MEb}^{+}$ estimate suffers an additional BC error compared to the *G*_MEb_ estimate, because of the differences between $\Theta_{a}^{s}$ and $\Theta_{e}^{s}$. The BC error of the $G_{MEb}^{+}$ estimate is similar to that of the ${\hat{G}}_{c}^{+}$ estimate in Section 5.3 (compare Figures 9 and 13). In particular, the BC error is at least as large as the information and forced‐transport errors for all metrics examined in Figure 13.

When all errors are considered, the $G_{MEb}^{+}$ estimate reconstructs the model truth with an error of 50% for basin integrated $H_{e}$ and 40% for zonal‐and‐depth integrated $\Delta H_{e}$ (Figures 13a–13d, RMSEs of orange lines). In the Indo‐Pacific the error is largest around 40°S, while in the Atlantic the error is of similar magnitude across latitudes (Figures 13c and 13d, compare black and orange lines). It is important to note that the *G*_MEb_ estimate is more accurate than the $G_{MEb}^{+}$ estimate for all metrics examined here. This highlights the need to reduce the BC error when applying inferred GFs to estimate the real‐world excess heat.

### 7.4 Sensitivity of the *G*_MEb_ Estimate

In this subsection, we examine how sensitive the *G*_MEb_ estimate is to the choice of data constraints and priors. For each sensitivity experiment, we focus on two metrics: (a) basin integral and (b) zonal‐and‐depth integrated change. Both metrics are calculated for $H_{e}$ only and integrated over the 0–2,000 m layers.

#### 7.4.1 Constraints From SF_6_ and Bomb Δ^14^C

In the first experiment, we add SF_6_ and bomb Δ^14^C at year 1994 as additional constraints, while keeping other settings unchanged. Adding SF_6_ alone or SF_6_ and Δ^14^C together has little impact on the *G*_MEb_ estimate (not shown). For instance, the RMSE change due to SF_6_ is less than 2% for all the metrics.

How much can SF_6_ and bomb Δ^14^C improve on the *G*_MEb_ estimate if it has little prior knowledge of ocean transports? We examine this question by replacing the FAMOUS prior in *G*_MEb_ with a uniform prior; the resulting *G*_ME_ is referred to as *G*_MEu_. The uniform prior is defined as

(32) $$\begin{array}{ccc} G_{pr}\left(r,0|r_{s},\tau \right) & = & \frac{1}{8,000A_{0}},0\geq \tau >-8,000\;\text{years},\\ G_{pr}\left(r,0|r_{s},\tau \right) & = & 0,\tau \leq -8,000\;\text{years}, \end{array}$$

where *A*_0_ is the surface area of the global ocean. The uniform prior assumes that a water parcel, regardless of its interior location, contains equal amounts of water from all surface locations and times over the previous 8,000 years. When the uniform prior is used, we relax the regularization parameter *λ* (see Section 6.2) from unity to 0.1 so that more modification to the prior is allowed compared to when the FAMOUS prior is used.

**Figure 14:**
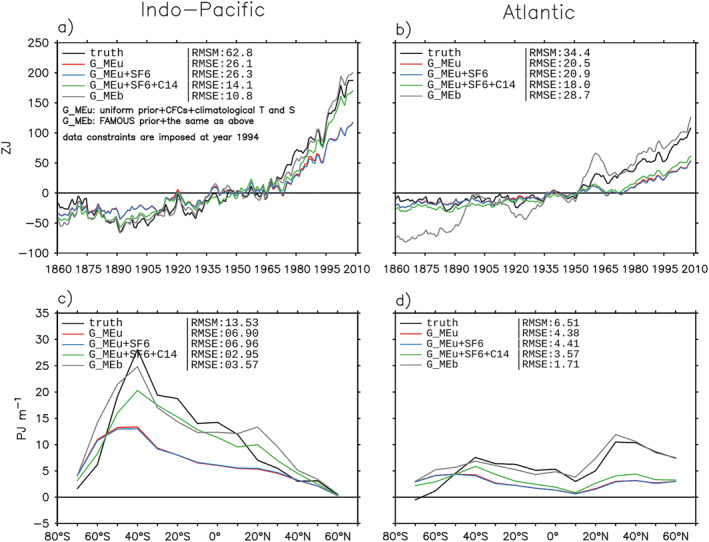
Sensitivity of the *G*_MEu_ estimate to additional constraints from SF_6_ and bomb Δ^14^C. (a and b) Basin integrated $H_{e}$. (c and d) Zonal‐and‐depth integrated $\Delta H_{e}$. The model truth is shown in black lines. The *G*_MEu_ estimates constrained by different tracers are color coded. For comparison, the *G*_MEb_ estimate is included as gray lines. The root‐mean‐square magnitude (RMSM) of the model truth and the root‐mean‐square errors (RMSEs) of different estimates are listed.

The *G*_MEu_ estimate of $H_{e}$ (red line) is about 50% lower than the *G*_MEb_ estimate (gray line) in both the Indo‐Pacific and the Atlantic (Figure 14). The *G*_MEu_ estimate is improved by adding bomb Δ^14^C (green line) as a constraint, but not by adding SF_6_ (blue line) (Figure 14). This is because bomb Δ^14^C and CFCs have very different surface BCs (Figures 10b and 10e), which provides additional constraints (equations) to the inverse problem. In contrast, the surface BCs are very similar between SF_6_ and CFCs. The improvement due to bomb Δ^14^C is much greater in the Indo‐Pacific than in the Atlantic. This is probably because the surface BCs of Θ_e_ and bomb Δ^14^C are more alike in the Indo‐Pacific than Atlantic (compare Figures 10e and 5d). In particular, the pattern of surface Θ_e_ peaks in the North Atlantic, whereas the pattern of surface bomb Δ^14^C is at its minimum in that region.

#### 7.4.2 Perturbing Prior GFs

In the second experiment, we replace the FAMOUS prior in the *G*_MEb_ estimate with Inverse Gaussian (IG) distributions of different shape, following Holzer et al. (2018). The IG distribution is the analytical form of *G*_c_ for constant 1D flow; a narrower IG distribution implies that the flow has a higher Peclet number (Waugh & Hall, 2002). The method of constructing the IG prior is described in Appendix E. The three IG priors tested here are called IG‐0.5, IG‐1.0, and IG‐1.5.

**Figure 15:**
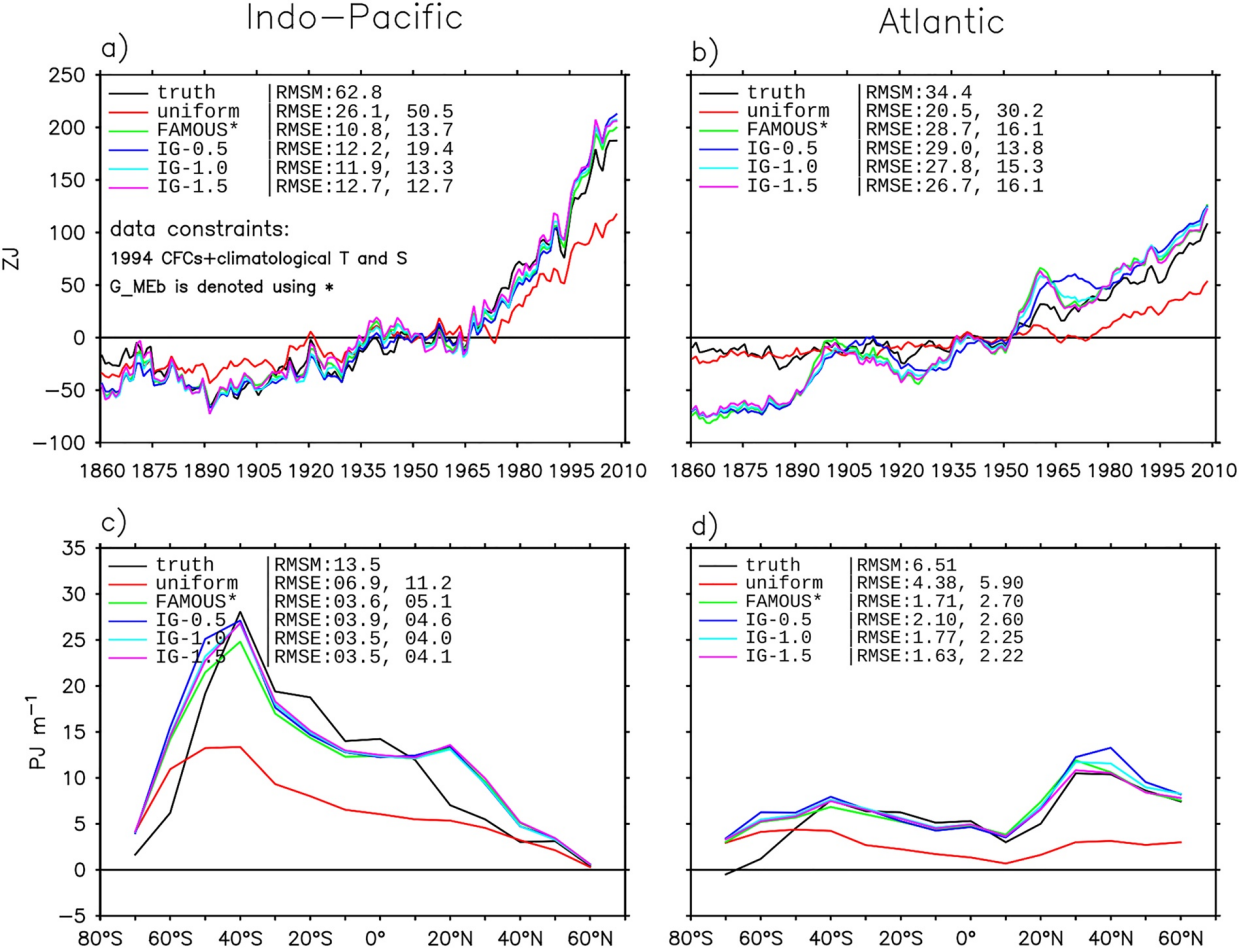
Sensitivity of the *G*_MEb_ estimate to the choice of prior (*G*_pr_). (a and b) Basin integrated $H_{e}$. (c and d) Zonal‐and‐depth integrated $\Delta H_{e}$. The model truth is shown in black lines. The *G*_MEb_ estimates with different *G*_pr_ are color coded. For each *G*_pr_, the RMSEs of the *G*_MEb_ and *G*_pr_ estimates are listed from left to right. IG‐0.5, IG‐1.0, and IG‐1.5 are approximations of the FAMOUS prior using Inverse Gaussian forms of different shape.

Replacing the FAMOUS prior with the IG priors leads to a change in RMSE of less than 20% for all metrics examined in Figure 15. Among the three IG priors, IG‐1.0 (corresponds to a Peclet number of one) gives the closest estimate compared to the FAMOUS prior. The RMSE of the *G*_MEb_ estimate (first column) is always reduced compared to that of the *G*_pr_ estimate (second column) regardless of which prior is used (Figure 15 numbers in the legends), except for the Atlantic integral. This highlights the constraints of CFCs and climatological temperature and salinity on the passage of excess heat in the ocean.

#### 7.4.3 Perturbing Time of Constraints

**Figure 16:**
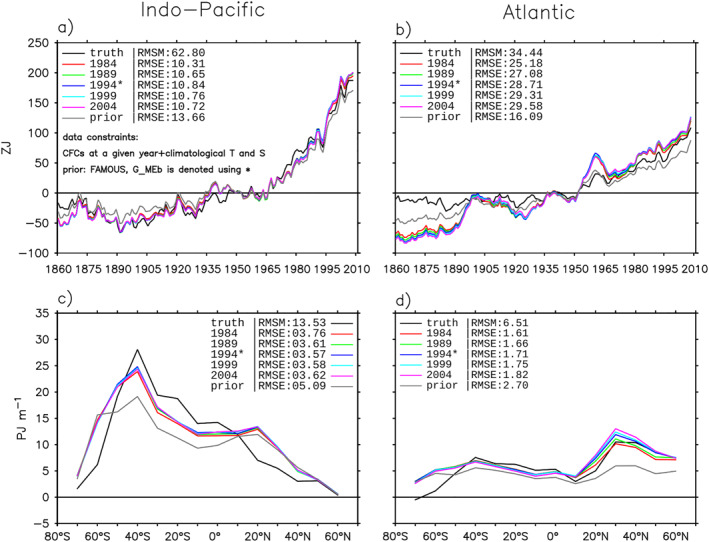
Sensitivity of the *G*_MEb_ estimate to the time of data constraints. (a and b) Basin integrated $H_{e}$. (c and d) Zonal‐and‐depth integrated $\Delta H_{e}$. The model truth is shown in black lines. The *G*_MEb_ estimates with different data years are color coded. The prior estimate is shown in gray lines. The root‐mean‐square magnitude (RMSM) of the model truth and the root‐mean‐square errors (RMSEs) of different estimates are listed.

In the third experiment, we alter the year of data constraints in the *G*_MEb_ estimate from 1994 to 1984, 1989, 1999, and 2004, while keeping other settings unchanged. The resulting change in RMSE is about 2% in the Indo‐Pacific and 5% in the Atlantic (Figure 16 RMSEs in the legends).

## 8 Summary

### 8.1 Excess Heat and Green's Functions

The ocean stores over 93% of the “excess heat” that has entered the climate system in recent decades (Meyssignac et al., 2019). This excess heat is added to the ocean surface by air‐sea fluxes (warming or cooling) and carried to depths by ocean transports. One method to estimate excess heat is to propagate its surface BCs downward using a GF representation of ocean transports. The GFs can be derived from: (a) simulating idealized tracers in a model (“simulated GFs”) or (b) solving an inverse problem using tracer observations (“inferred GFs”) (Holzer et al., 2010; Khatiwala et al., 2009; Zanna et al., 2019). The BCs are often derived from SST anomaly in the literature.

### 8.2 Errors in the GF Method

The GF estimate of excess heat is inaccurate for the following reasons.

1. Patch error: Simulated GFs are coarse grained in space and time, hence they partially neglect the covariance between the true GFs and surface BCs.
2. Transport error: Simulated/inferred GFs do not resolve time‐varying ocean transports due to unforced variability and forced change.
3. Information error: Observations are insufficient constraints for inferring GFs.
4. BC error: SST anomalies are contaminated by redistributed changes.
5. Model error: Modeled ocean transports encoded in simulated GFs are different from those of the real world.

### 8.3 HadCM3 Perfect‐Model Test

How different errors affect the accuracy of the GF method has not been examined in the literature. Here, we investigate this question using a historical simulation (1860–2008) conducted in the HadCM3 AOGCM. We treat this simulation as the real world, and compare excess heat $H_{e}$ diagnosed in it (as the “truth”) with that estimated using simulated/inferred GFs. Details on how different errors are computed are given in Sections 4.4 and 7.1. We focus on evaluating $H_{e}$ derived from GFs instead of GFs themselves, because not every detail in GFs matters for estimating $H_{e}$.

### 8.4 Estimating Excess Heat Using Simulated GFs

We generate simulated GFs in a 200‐year pre‐industrial control experiment of HadCM3.

#### 8.4.1 How Accurate Is the Method?

The simulated GFs reconstruct $H_{e}$ in the Indo‐Pacific with a RMS error of 48% for the volume integral and 26% for zonal‐and‐depth integrated changes; the corresponding numbers are 39% and 37% in the Atlantic, respectively (including all errors except the model error). The volume integral is most affected by the forced‐transport and BC errors; the patch error is <1/3 of the BC error in terms of the RMS value. The zonal‐and‐depth integral is affected by the patch, forced‐transport and BC errors to a similar degree; the BC error is slightly larger than the other two in the Indo‐Pacific. The unforced‐transport error is <1/3 of the patch error for all metrics examined here. Results of this subsection are summarized in Figure 9.

#### 8.4.2 Underestimated or Overestimated?

The patch error causes an underestimate of $H_{e}$ in the North Atlantic, and an overestimate of $H_{e}$ south of 40°S. The forced‐transport error causes an overestimate of $H_{e}$ at most latitudes, especially in the northern subtropics. The BC error causes an underestimate of $H_{e}$ in the Southern Ocean. This underestimate partially cancels out the patch and forced‐transport errors, reducing the total error in the Southern Ocean. Note that the degree to which this error compensation would work may be different in the real world and in other models.

### 8.5 Estimating Excess Heat Using Inferred GFs

We compute inferred GFs by using HadCM3 equivalents of the GLODAPv1 data as constraints to update a prior estimate of GFs. The GLODAPv1 data consist of CFC‐11 and CFC‐12 at year 1994 and climatological temperature and salinity (Key et al., 2004).

#### 8.5.1 How Accurate Is the Method?

The inferred GFs reconstruct $H_{e}$ in the Indo‐Pacific with an error of 50% for the volume integral and 34% for zonal‐and‐depth integrated changes; the corresponding numbers are 44% and 42% in the Atlantic, respectively (including all errors). The volume integral is most affected by the BC error; the information and forced‐transport errors are about 2/3 of the BC error (in terms of the RMS value) in the Indo‐Pacific. The zonal‐and‐depth integral is affected by the information, forced‐transport and BC errors to a similar degree; although the BC error is slightly larger than the other two in the Indo‐Pacific. Results of this subsection are summarized in Figure 13.

#### 8.5.2 Underestimated or Overestimated?

The information error causes an underestimate of $H_{e}$ at most latitudes (except south of 50°S). The forced‐transport and BC errors have the same effects as discussed with simulated GFs. In the Atlantic, the information error partially compensates the forced‐transport error, reducing the total error there. It is unclear whether the same compensation would occur in the real world or in other models. Removing the BC error improves the estimate with inferred GFs significantly.

#### 8.5.3 Sensitivity to Data Constraints and Priors

The estimate of $H_{e}$ from inferred GFs is not sensitive to: (a) shifting the data year by ±10, (b) small changes in the shape of prior GFs, or (c) adding 1994 SF_6_ and bomb ^14^C as additional constraints, although bomb ^14^C (but not SF_6_) helps when a less informative prior is used.

## 9 Discussions

### 9.1 Model Error of Simulated GFs

Because we use GFs simulated in HadCM3 to estimate excess heat $H_{e}$ in the HadCM3 world, our results do not include the model error. To explore this error, one could perturb simulated GFs to generate an ensemble of estimates (Zanna et al., 2019). An alternative would be to use more than one AOGCM; by treating one of them as though it were perfect, one could make an estimate with the GFs of another. This approach would not include the effect of errors common to all models.

### 9.2 Is Air‐Sea Flux GF a Better Option?

As well as concentration BCs, one can propagate surface heat fluxes to estimate $H_{e}$ using simulated GFs (with a different configuration). In HadCM3, we find that this method gives a better estimate of $H_{e}$ than propagating concentration BCs. However, observations of surface heat flux are not adequate for the purpose of estimating $H_{e}$. For example, the Objectively Analyzed air‐sea Fluxes (Yu et al., 2008) are not available before 1985 and do not have the accuracy to resolve the global mean energy imbalance.

### 9.3 Simulated GFs Versus Ocean Model

Evolution of passive tracers in the model world can be studied using simulated GFs as well as ocean models. Ocean models are more accurate than simulated GFs for this regard, because they do not suffer the patch and transport errors. In addition, GFs are computationally expensive to derive.

Nonetheless, simulated GFs are useful for the following purposes. First, simulated GFs encapsulate the effect of a model's ocean transports in a form that can be easily shared within the community. Especially, GFs are much easier to use than 3D ocean models. Second, simulated GFs can be used to quantify the surface sources and timescales of a tracer response (e.g., Marzocchi et al., 2021; Wu et al., 2021; Zanna et al., 2019).

### 9.4 Improving Simulated GFs

Simulating GFs with finer surface patches can reduce the patch error. At the limit that every grid box is a patch, the patch error is completely eliminated. What is the best strategy to simulate GFs given a limited amount of computer time? Air‐sea fluxes and surface concentrations of a tracer often exhibit low‐dimensional structures in space. Designing patches around these structures can reduce the patch error at low computational cost (see Appendix F for two examples). On the time dimension, simulating GFs starting from various years in a historical simulation (e.g., Marzocchi et al., 2021) can reduce the unforced‐ and forced‐transport errors. For instance, a set of GFs per year can capture time variation of ocean transports on interannual and longer timescales. Simulating GFs starting from every 10 years of the historical run would be less accurate, but more appealing computationally. Sensitivity tests to find the optimal time interval for time‐dependent GFs would be useful.

### 9.5 Improving Inferred GFs

To reduce the information error, one could add observations in the GLODAPv2 data set (Olsen et al., 2016) as additional constraints. At present, CFCs only constrain GFs at multi‐decadal and shorter lead times, limited by their surface histories. It is important to maintain observations of transient tracers in the ocean, so that new observations can be added to constrain GFs over longer lead times in the future.

### 9.6 Excess Temperature BCs

To derive excess temperature BCs, one could combine modeled patterns of surface excess temperature and observed global‐mean SST anomalies to form hybrid excess temperature BCs. These new BCs may help reduce the contamination of redistributive cooling in the SST BCs (e.g., Figure 5). Note that there are uncertainties in the modeled patterns of excess temperature because of the spread in the modeled surface heat fluxes (e.g., in the CMIP6 ensemble).

**Notation**
**r**
: 3D position vector of the ocean
**r**_s_
: 2D position vector of the ocean surface
*t*
: time variable in general
*t*_s_
: time variable of surface source
*Q*_ctrl_
: Net surface heat fluxes in the pre‐industrial control experiment
*Q*_ERF_
: Effective radiative forcing of the historical experiment
*Q*′: Changes in surface heat fluxes due to climate feedbacks
Θ_ctrl_
: Ocean potential temperature in the pre‐industrial control experiment
Θ_hist_
: Ocean potential temperature in the historical experiment
Φ: 3D ocean transport operator in general
*L*_ctrl_,Φ: saved from the pre‐industrial control experiment
*L*_hist_,Φ: saved from the historical experiment
Θ_a_
: Historical ocean temperature anomaly, Θ_a_ = Θ_hist_ − Θ_hist_
Θ_e_
: Excess temperature tracer evolved by *L*_hist_
$\Theta_{e}^{*}$
: Excess temperature tracer evolved by *L*_ctrl_
Θ_r_
: Redistributed temperature tracer, Θ_a_ = Θ_e_ + Θ_r_
$H_{a}$
: Historical ocean heat content anomaly, extensive form of Θ_a_
$H_{e}$
: Excess heat content evolved by *L*_hist_, extensive form of Θ_e_
$H_{e}^{*}$
: Excess heat content evolved by *L*_ctrl_, extensive form of $\Theta_{e}^{*}$
$H_{r}$
: Redistributed heat content, extensive form of Θ_r_
*X*
: Concentration of a tracer, *X* could be Θ_a_, Θ_e_, $\Theta_{e}^{*}$, etc.
*X* ^s^
: *X* at the surface
${\hat{X}}^{s}$
: *X* ^s^ averaged onto a yearly grid and surface patches
*G*
: Boundary GF of tracer transport equation in general
*G*_c_
: *G* defined for surface concentration BCs
*G*_f_
: *G* defined for air‐sea tracer fluxes or surface sources/sinks
${\hat{G}}_{c}$
: Simulated *G*_c_ defined for yearly‐ and patch‐averaged surface conditions
${\hat{G}}_{f}$
: Simulated *G*_f_ defined for yearly‐ and patch‐averaged surface sources/sinks
*G*_ME_
: Maximum entropy estimate of *G*_c_ in general
*G*_pr_
: Prior estimate of *G*_c_ used in *G*_ME_
*G*_MEb_
: *G*_ME_ constrained by GLODAPv1 data and the FAMOUS prior
*G*_MEu_, *G*_ME_
: constrained by GLODAPv1 data and the uniform prior

**Acronyms**
GF: Green's function
BC: boundary condition
MaxEnt: maximum entropy
IG: Inverse Gaussian
RMSE: root‐mean‐square error
RMSM: root‐mean‐square magnitude

## Data Availability

Outputs from the historical simulation are published at https://doi.org/10.5281/zenodo.6790458. Simulated tracer Green's functions are published at https://doi.org/10.5281/zenodo.6792335. The CESM2 data are available at https://esgf-node.llnl.gov. For the use of the HadCM3 model, contact UM_collaboration@metoffice.gov.uk.

## Appendix A Forcing the Historical Simulation

We did not conduct the historical simulation by prescribing CO_2_ and other forcing agents in the atmosphere. This is because HadCM3 does not include an up‐to‐date treatment of anthropogenic aerosol forcing, especially aerosol‐cloud interaction. (In the historical experiments of Stott et al. (2000), the effect of tropospheric aerosol on cloud reflectivity was approximately represented by prescribed perturbations to surface albedo.) In addition, since our focus is on heat uptake, it is convenient to prescribe the heat flux which is added to the ocean. The use of surface forcing omits any non‐radiative effects of forcing agents and their radiative effects directly in the atmosphere. But those effects are relatively minor and not important in this work. As shown in Section 2.3, the historical simulation is sufficiently close to observations for the purposes of this study.

## Appendix B Passive and Dynamical Θ_e_ Definitions

In Section 2, we define excess temperature Θ_e_ as a passive tracer evolved by the pre‐computed transport operator *L*_hist_ (Equation 6). Alternatively, we can define Θ_e_ as a dynamical tracer that affects ocean transports.

(B1)
$$\begin{array}{ll} & \frac{\partial \Theta_{e}}{\partial t}+\Phi \left(\Theta_{e}\right)=\frac{1}{\rho_{0}c_{p}dz_{1}}\left(Q_{\text{ERF}}+Q^{\prime }\right),\\ & \text{initial condition:}\Theta_{e}\left(0\right)=0. \end{array}$$

Φ is the ocean transport operator in general (Equation 2). Φ(Θ_e_) is a nonlinear function of Θ_e_ because Θ_e_ affects Θ which affects Φ. Integrating Equation B1 in the historical simulation gives the same evolution of Θ_e_ as that derived from Equation 6. The two definitions, however, correspond to different constructions of GFs.

The GFs of Equation 6 are “passive” GFs; they are evolved by the same *L*_hist_ operator (see Equation 14) regardless of the time and location of the Θ_e_ perturbation, because *L*_hist_ is a pre‐computed quantity. To simulate GFs for Equation 6 one only needs pre‐computed velocities and diffusivities in principle. In contrast, the GFs of Equation B1 are “dynamical” GFs; they are evolved by different Φ operators, because Φ depends on the location and time of the Θ_e_ perturbation. To simulate GFs for Equation B1 one needs a full ocean model to interactively compute Φ for every Θ_e_ perturbation.

Because Φ depends on the forcing, the solution of Equation B1 cannot be written as a superposition of impulse responses scaled with the forcing. That is, the GF estimate of the dynamical Θ_e_ has a nonlinear error. The GF estimate of the passive Θ_e_, however, does not have a nonlinear error, because *L*_hist_ is independent of the forcing (i.e., Equation 6 is strictly linear). The unforced‐ and forced‐transport errors (Section 4.1) arise because we neglect the time‐evolution of GFs to reduce computational time, not because the GF method is inherently inaccurate.

## Appendix C Maximum Entropy Method

### C1 Problem Formulation

Inferring *G*_c_(**r**, 0 | **r**_s_, *τ*) from *X*_*n*_(**r**, *t*_*n*_) and $X_{n}^{s}\left(r_{s},t_{s}\right)$ is an underdetermined problem. Among infinitely many estimates of *G*_c_ that satisfy Equation 23, we choose one based on the principle of Minimum Discrimination Information (MDI) (Kullback, 1959). Recall that *G*_c_ is like a Probability Distribution Function (PDF) (Section 3). The principle of MDI states that: to update a prior PDF using new facts (i.e., constraints), the new PDF should be chosen such that it is the “nearest” to the prior PDF. The distance between two PDFs is measured using relative entropy in MDI.

Applying the principle of MDI, we determine the best estimate of *G*_c_ by solving the following constrained optimization problem. The Lagrangian function of the problem is given as

(C1)
$$L\left(G,a_{1},\cdots ,a_{N}\right)=D_{\text{KL}}\left(G|G_{pr}\right)+\sum_{n=1}^{N}a_{n}\left(X_{n}\left(r,t_{n}\right)-X_{n}^{\prime }\left(r,t_{n}\right)\right).$$

*G* is an unknown PDF. *G*_pr_ is a prior PDF. *D*_KL_(*G* | *G*_pr_) is the Kullback‐Leibler divergence, or the relative entropy, between *G* and *G*_pr_

(C2)
$$D_{\text{KL}}\left(G|G_{pr}\right)=\int_{\Omega }d^{2}r_{s}\int_{-\infty }^{0}G\left(r,0|r_{s},\tau \right)\log\frac{G\left(r,0|r_{s},\tau \right)}{G_{pr}\left(r,0|r_{s},\tau \right)}d\tau .$$

*a*_*n*_ is the Lagrange multiplier. *N* is the total number of constraints. Each constraint takes the form $X_{n}=X_{n}^{\prime }$. $X_{n}^{\prime }$ is an estimate of *X*_*n*_ using *G*, calculated by substituting *G* into Equation 23.

(C3)
$$X_{n}^{\prime }\left(r,t_{n}\right)=\int_{\Omega }d^{2}r_{s}\int_{-\infty }^{t_{n}}G\left(r,0|r_{s},t_{s}-t_{n}\right)X_{n}^{s}\left(r_{s},t_{s}\right)dt_{s}.$$

### C2 Exact Solution

The optimization problem is solved by setting ∂L/∂G=0 and $\partial L/\partial a_{n}=0$ simultaneously. Because L is a function of the function *G* (i.e., L is a functional), we use the Euler‐Lagrange equation to solve ∂L/∂G=0. First, we substitute Equations C2 and C3 into Equation C1 and rewrite L as

(C4)
$$L\left(G,a_{1},\cdots ,a_{N}\right)=\int_{\Omega }d^{2}r_{s}\int_{-\infty }^{0}f\left(r_{s},\tau ,a_{1},\cdots ,a_{N}\right)d\tau +\sum_{n=1}^{N}a_{n}X_{n}\left(r,t_{n}\right),$$

where

(C5)
$$f=G\left(r,0|r_{s},\tau \right)\log\frac{G\left(r,0|r_{s},\tau \right)}{G_{pr}\left(r,0|r_{s},\tau \right)}-\sum_{n=1}^{N}a_{n}G\left(r,0|r_{s},\tau \right)X_{n}^{s}\left(r_{s},\tau +t_{n}\right).$$

Next, we solve *∂f*/*∂G* = 0. This yields *G*_ME_ in Equation 24, where *G*_ME_ is a function of *N* unknowns *a*_1_, ⋯, *a*_*N*_. Substituting *G*_ME_ into Equation 23 turns $X_{n}^{\prime }$ into a function of the *N* unknowns *a*_1_, ⋯, *a*_*N*_ as well. Finally, *a*_1_, ⋯, *a*_*N*_ are determined by using *N* constraint equations derived from setting $\partial L/\partial a_{n}=0$. Collecting *X*_*n*_, $X_{n}^{\prime }$ and *a*_*n*_ into column vectors **x**, **x**′, and **a**, respectively, the *N* constraint equations can be written as

(C6)
$$x^{\prime }\left(a\right)=x,$$

where **a** can be solved using standard numerical routines, for example, fsolve in MATLAB.

The principle of MDI is also known as the principle of Maximum Entropy (MaxEnt) (Jaynes, 1957), which maximizes the negative relative entropy subject to constraints. Here, we refer to the procedure of deriving Equation 24 as the MaxEnt method following Khatiwala et al. (2009).

### C3 Least Squares Solution

In practice, an exact fit between *X*_*n*_ and $X_{n}^{\prime }$ (i.e., Equation C6) is not desirable, because there are errors in Equation 23 and in observations. Solving for **a** using Equation C6 sometimes results in an overfitted *G*_ME_ that is difficult to interpret physically. For instance, a *G*_ME_ may have extremely large values at just a few **r**_s_ and *τ* locations, as opposed to a much broader distribution in *G*_pr_ derived from models. These extreme cases often come with large *a*_*n*_ values, which modify *G*_pr_ via the exponential function in Equation 24.

To avoid overfitting, we relax the equality constraints and solve for **a** in a least squares sense with Tikhonov regularization (Tikhonov & Arsenin, 1977). This gives *a*_*n*_ in Equation 26. The regularization term $\lambda \|a{\|}_{2}^{2}$ penalizes large *a*_*n*_ values that cause *G*_ME_ to deviate from *G*_pr_ substantially.

We determine the *λ* value using the L‐curve method (Hansen & O’Leary, 1993). A smaller *λ* value corresponds to a smaller model‐data misfit and a larger $\|a{\|}_{2}^{2}$ value. Setting *λ* = 0 gives **a** without the regularization (i.e., Equation C6). The L‐curve method finds the smallest *λ* allowed before any further decrease of *λ* leads to a rapid increase of $\|a{\|}_{2}^{2}$. The L‐curve method requires repeating the minimization process for different *λ* values, which is computationally prohibitive if carried out at each interior location. We choose *λ* by applying the L‐curve method to the centers of the subtropical gyres, where ocean tracers tend to accumulate. We find that the optimal *λ* value is between 0.1 and 10 in those locations; *λ* values within that range give a similar model‐data misfit and a similar $\|a{\|}_{2}^{2}$ value. Based on this evidence, we set *λ* to unity globally in this study.

## Appendix D Simulating Tracer Observations

We include CFCs, SF_6_ and bomb Δ^14^C in the HadCM3 historical simulation. All these tracers are simulated in the ocean with zero initial conditions and prescribed surface concentration boundary conditions (BCs) from 1860 to 2008. The BCs are derived by interpolating monthly outputs of the CESM2 historical simulation to each timestep of HadCM3 linearly. CESM2 is chosen here because it is the only model available to us that includes CFCs, SF_6_ and Δ^14^C in a historical simulation.

For simplicity, we choose not to simulate the air‐sea gas transfer of chemical tracers in HadCM3, which is different from the CMIP6 biogeochemical protocol (Orr et al., 2017). This is because the MaxEnt method is not concerned with the air‐sea gas transfer. *G*_ME_ is determined by the relationship between $X_{n}^{s}$ and *X*_*n*_, which is only affected by ocean transports (e.g., *L*_hist_ or *L*_ctrl_).

The radioactive decay of ^14^C can be accounted for by adding an exponential decay term in Equation 23 (see Holzer et al., 2010). Because the history of bomb Δ^14^C is very short compared to its half‐life, we neglect its radioactive decay in this study. The method of simulating Δ^14^C (hence the ^14^C/^12^C ratio) as a tracer was first proposed by Toggweiler et al. (1989).

## Appendix E Inverse Gaussian Prior

Following Waugh and Hall (2002) the Inverse Gaussian (IG) distribution is given as

(E1)
$$IG\left(\tau ,\Gamma ,\Lambda \right)=\sqrt{\frac{\Gamma^{3}}{4\pi \Lambda^{2}\tau^{3}}}\exp\left(-\frac{\Gamma {\left(\tau -\Gamma \right)}^{2}}{4\Lambda^{2}\tau }\right)\text{for}\;\tau >0.$$

*Γ* > 0, Λ > 0, and Γ^3^/(2Λ^2^) are the mean, the width and the shape parameter of the IG distribution, respectively. Equation E1 is the analytical form of *G*_c_ for 1D flow with constant velocity and diffusivity; Γ^2^/Λ^2^ is the Peclet number of the flow (Waugh & Hall, 2002).

We generate an ensemble of IG priors by fitting a scaled IG distribution to the FAMOUS prior (Equation 27) with varying Λ/Γ ratios.

(E2)
$$G_{pr}\left(r,0|r_{s},\tau \right)=b\left(r,r_{s}\right)IG\left(-\tau ,\Gamma ,\Lambda \right)$$

A higher Λ/Γ ratio corresponds to a more diffusive flow, in which water parcels are ventilated over a wider range of timescales. The Λ/Γ ratio is zero for pure advective flow.

For every **r**_s_ and a given Λ/Γ ratio, we choose Γ such that the resulting IG prior has the same mean age as the FAMOUS prior for 0 ≥ *τ* > −1,000 years. The mean age between *τ*_1_ and *τ*_2_ is calculated as

(E3)
$$\left(\sum_{\tau =\tau_{1}}^{\tau_{2}}\tau G\left(r,0|r_{s},\tau \right)\right)/\left(\sum_{\tau =\tau_{1}}^{\tau_{2}}G\left(r,0|r_{s},\tau \right)\right).$$

*b*(**r**, **r**_s_) determines the fraction of water at **r** that is formed at **r**_s_ over all *τ* values (i.e., the denominator of Equation E3); we set this parameter to be the same as the one in the FAMOUS prior.

We generate the IG prior for the Λ/Γ ratio of 0.5, 1.0, and 1.5 to cover its likely range in the ocean (Waugh et al., 2006). These priors are referred to as IG‐0.5, IG‐1.0, and IG‐1.5, respectively.

## Appendix F Alternative Patch Designs

Instead of decomposing BCs into pulses in the lon‐lat space, one could project BCs onto Empirical Orthogonal Functions (EOFs), and construct GFs based on the leading EOFs. (EOFs are the optimal coordinates to capture the spacetime variability of a field.) This method has one limitation: the sources of a tracer are often interpreted in terms of water‐mass formation sites, but EOFs do not always project back to isolated regions in the lon‐lat space.

One could also reduce the patch error by prescribing a spatial pattern within every patch when simulating GFs. Such a pattern can be derived from long‐term trends for surface excess temperature or long‐term averages for surface heat fluxes, for instance. In this way, the covariance between the true GFs and BCs are better incorporated into simulated GFs than assuming that BCs are uniform within every patch. However, because tracers exhibit different patterns in their BCs, there is no universal pattern that would work for every tracer.
